# Decoupling Dynamics and Crosslink Stability in Supramolecular Hydrogels Using Associative Exchange

**DOI:** 10.1002/adma.202516741

**Published:** 2026-01-22

**Authors:** Pierre Le Bourdonnec, Charafeddine Ferkous, Léo Comunale, Luca Cipelletti, Rémi Merindol

**Affiliations:** ^1^ Laboratoire Charles Coulomb CNRS Université de Montpellier Montpellier France; ^2^ Institut Universitaire De France Paris France

**Keywords:** associative reorganizations, DNA hydrogels, relaxations, rheology, rupture mechanics, supramolecular networks

## Abstract

The design of hydrogels that combine mechanical robustness with dynamic reconfigurability remains a fundamental challenge, as increasing crosslink dissociation rates compromise network integrity. This limitation is addressed through the incorporation of an associative crosslink exchange into DNA‐based supramolecular hydrogels, enabling the decoupling of network relaxation behavior from crosslink stability. The hydrogels are constructed from enzyme‐synthesized single‐stranded DNA that self‐assembles via hybridization between complementary domains. These crosslinks can reorganize through dissociative melting or associative strand displacement reaction, yielding networks with tunable relaxation timescales spanning over three orders of magnitude. Rheological measurements and thermodynamic modeling confirm that associative exchange facilitates efficient stress dissipation without diminishing rupture strength or thermal stability. In contrast, dissociative systems inherently trade increased dynamics with mechanical weakening. This decoupling is achieved through the implementation of a catalytic reorganization pathway governed by the composition of the sample, independently of the crosslink strength. These findings establish the mechanism of reorganization as a key design parameter for engineering adaptive soft materials that combine resilience and responsiveness.

## Graphical abstract

How does reorganization impact the stability of supramolecular hydrogels? In contrast to dissociative crosslink exchange, implementing associative exchange in macroscale DNA hydrogels enables decoupling of reorganization dynamics from thermal and mechanical stability. This establishes the reorganization mechanism, and its underlying thermodynamic pathway, as a key design parameter for creating robust, reconfigurable soft materials.

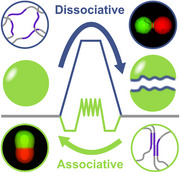

## 1 Introduction

Inspired by living tissues, hydrogels are water‐swollen polymer networks that possess unique abilities to interact with biological systems. There is a growing interest in developing hydrogels for drug delivery, cartilage replacement, biomedical devices, and as artificial extracellular matrices [1, 2, 3]. Producing synthetic hydrogels with mechanical behaviors that closely mimic those of biological materials is of prime interest to facilitate biological interfacing. For implants and cartilage replacement, the focus is on designing strong and tough hydrogels capable of resisting fracture [4, 5]. For cell culture, the emphasis is on engineering dynamic hydrogels that can replicate the cellular microenvironment [6, 7]. We already know that altering the elastic modulus [8, 9, 10], stress relaxation [11, 12], and stress stiffening properties [13, 14, 15] of the surrounding matrix has a profound impact on cell behavior. Surprisingly, biological tissues that consist of supramolecular networks, are both dynamic and tough []. These two properties are often mutually exclusive in synthetic systems, highlighting a gap in our understanding of how to decouple mechanical strength and dynamic behavior [17].

Several key molecular parameters for the rational design of advanced hydrogels have already been identified. On the one hand, tailoring the network architecture primarily governs hydrogel elasticity [18]. Beyond linear mechanics, some of the most elegant structural designs incorporate slide‐ring crosslinks, ideal network architectures, or interpenetrating networks to enhance ultimate stress and toughness [19, 20, 21]. On the other hand, dynamic crosslinking chemistries enable internal reorganization within the hydrogel network. Many strategies have been explored, including coordination bonds [22, 23], host–guest interactions [24], dynamic covalent bonds [25, 26, 27], and oligonucleotide self‐assembly [28, 29, 30]. In such materials, the characteristic lifetime of the dynamic bonds dictates the network's relaxation behavior. In principle, the network architecture and the lifetime of the crosslinks can be tuned independently to control the network's linear elasticity and relaxation time, respectively [22, 30]. However, changing the lifetime of the crosslinks, hence their dissociation rate, may also impact the equilibrium concentration of associated/dissociated crosslinks, which in turns impact the network architecture [31]. Furthermore, a fundamental trade‐off persists between the strength and the reorganization speed of hydrogels [32]. In networks where dissociation is thermally driven, thermal agitation imposes an upper limit on the binding energy of the crosslinks, which in turns limits the strength of a dynamic network, a concept summarized as “strong means slow” [33]. Overcoming this limitation requires moving away from thermally driven dissociation and introducing a new design parameter: The reorganization mechanism.

The conflict between bond strength and dynamics in hydrogels mirrors a similar trade‐off in bulk polymers. Thermosets are mechanically stable but cannot be recycled, whereas thermoplastics are reprocessable but suffer from creep and solubility in good solvents. Embedding catalytic reorganization mechanisms can address this challenge by enabling covalent bond rearrangement [34]. In particular, vitrimers, materials that undergo associative crosslink exchange, have gained significant attention [35, 36, 37]. Associative exchange involves an intermediate state where bond formation precedes bond dissociation, allowing melding and recycling of vitrimers without compromising on their strength and stability. However, due to the hydrolytic sensitivity of most vitrimer chemistries, associative crosslink exchange remains underexplored in hydrogels [38, 39, 40]. As a result, we lack a clear understanding of how the reorganization mechanism, either associative or dissociative, governs the macroscopic behavior of hydrogels. In this work, we propose to transfer the concepts developed for bulk polymers, into supramolecular hydrogels, using DNA, in order to explore the impact of the reorganization mechanisms onto the macroscale mechanical behavior.

Beyond the storage of genetic information, DNA self‐assembly offers one of the most powerful platforms for designing custom supramolecular materials. The hybridization of complementary DNA strands enables precise control over nanoscale architectures [41, 42], binding energies [43, 44, 45], and even reorganization kinetics via strand displacement reactions [46, 47, 48]. Consequently, DNA has emerged as a platform of choice for engineering dynamics, responsive, and biocompatible hydrogels [49, 50, 51]. DNA crosslinks are inherently dissociative: when sufficient thermal energy is applied, the hydrogen bonds between complementary strands break, leading to crosslink rupture [52]. Yet, associative crosslink exchange can be programmed using strand displacement reactions [29, 53]. This makes DNA hydrogels an ideal system to systematically investigate how reorganization mechanisms influence mechanical behavior. So far, however, this could not be tested in macroscale samples: systematic rheological studies of macroscale DNA hydrogels are rare, largely due to the high cost of synthetic oligomers. We overcome this limitation using rolling circle amplification (RCA), an isothermal enzymatic process that produces milligram quantities of sequence‐controlled DNA and facilitates hydrogel assembly [50, 54]. In this article we use RCA to program associative and dissociative supramolecular reorganization mechanisms into macroscale hydrogels and explore their impact on the hydrogel mechanical behavior.

It may seem counterintuitive to implement an associative crosslink exchange, akin to the one found in vitrimers, in a non‐covalent network. Arguably, a key feature of vitrimers is to enable reprocessability, specifically in covalent networks. Here we aim to show that, even in non‐covalent networks, the implementation of an associative exchange mechanism driven by defined supramolecular self‐assemblies has a profound impact on the macroscale behavior of a hydrogel. In particular, because we rely on custom DNA constructs with predictable self‐assembly characteristics, we can rationalize the macroscale mechanical behavior of the hydrogels from a thermodynamic perspective. In the linear regime, we show that associative crosslink exchange allows controlling reorganization at temperatures that are well below melting. More importantly, we show that in the non‐linear regime such control over the reorganization mechanism allows for decoupling the rupture strength from reorganization speed. Overall, this work demonstrates the key role of the reorganization mechanism in supramolecular, non‐covalent networks.

## 2 Assembly of Dynamic DNA Hydrogels

**FIGURE 1:**
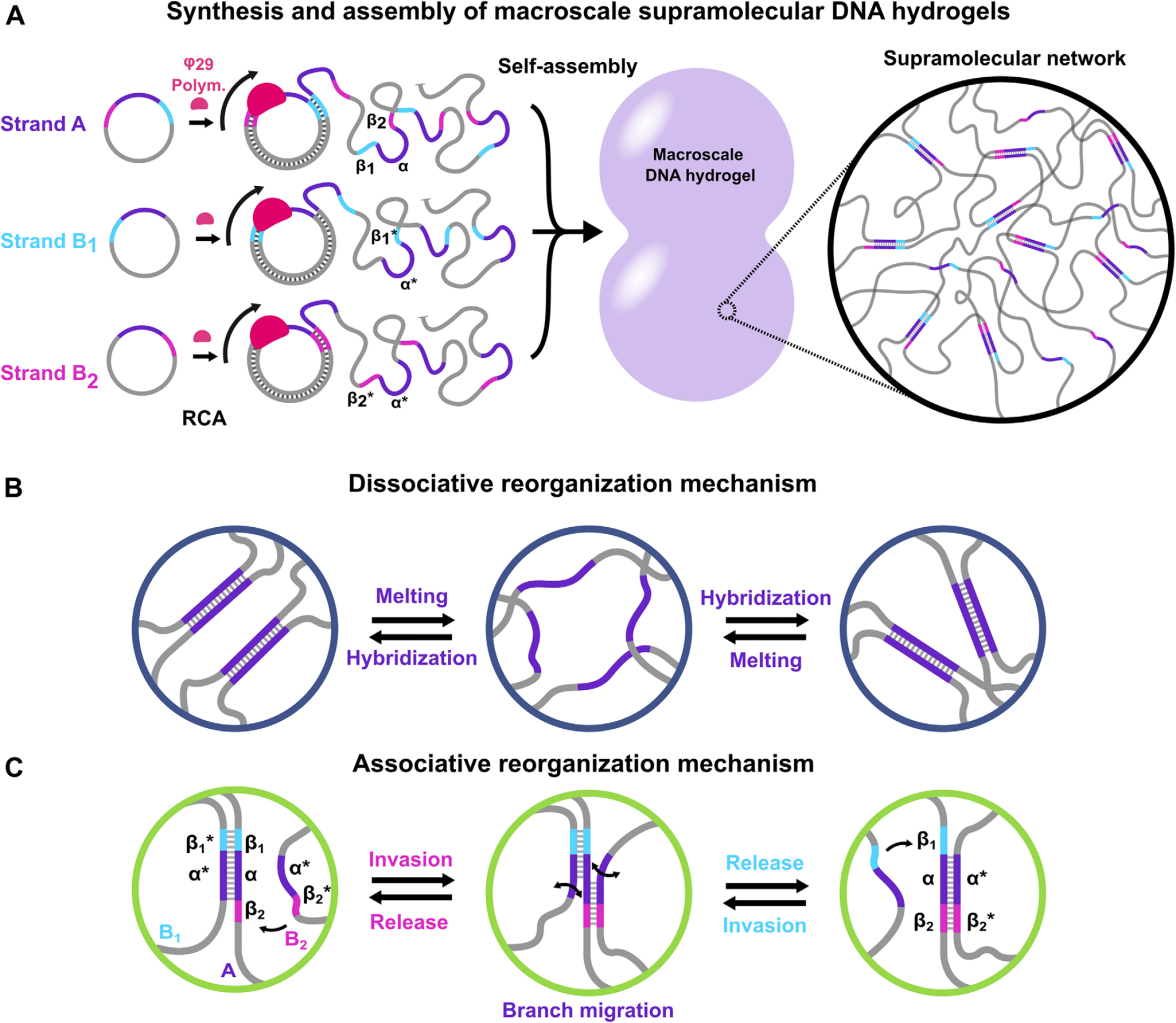
(A) Schematic representation of the DNA strands synthesized by rolling circle amplification (RCA) and the network architecture of the macroscale supramolecular DNA hydrogels resulting from their assembly. (B,C) Key mechanisms controlling crosslink reorganization. (B) Dissociative reorganization driven by the melting and re‐hybridization of a crosslinking duplex. (C) Associative exchange driven by the invasion, branch migration, and release of a crosslinking duplex (i.e., a strand displacement reaction).

The DNA synthesis and hydrogel assembly are presented in Figure 1. The enzymatic synthesis (RCA) uses a small circular template and nucleoside triphosphates as inputs and yields long single‐stranded DNA consisting of multiple copies of the template linked one after another (see also Figure S1 for details). Macroscale hydrogels are formed by mixing RCA products with complementary domains (e.g., α and α^⁎^). After heating above 95°C to melt all DNA duplexes and cooling down to room temperature, we obtain a homogeneous supramolecular network held together by DNA duplexes (see also Figure S2 for details) [50]. Such a supramolecular DNA hydrogel is dissociative (Figure 1B). When provided with sufficient thermal energy, the complementary domains can spontaneously melt and rehybridize, allowing network reorganization. Such dissociative reorganization is common in supramolecular networks [17, 22, 24, 52].

Here, we further engineer an associative exchange using a strand displacement reaction programmed into the repeat sequences of three RCA products: A pivot strand A and two exchange strands B_1_ and B_2_ (Table 1 and Figure 1C). The repeat sequence of the pivot strand consists of a crosslink domain α, flanked by two toeholds domains β_1_ and β_2_. The repeat sequences of the exchange strands consist of a crosslink‐complementary domain α^⁎^ flanked by one of the toehold‐complementary domains, either β_1_^⁎^ or β_2_^⁎^. For each strand, the functional domains are separated on the RCA product by an adenine spacer (A_30_), which provides flexibility. Upon mixing, the crosslink domains (α and α^⁎^) of the exchange and pivot strands hybridize, forming the 3D network. These sequences also enable associative exchange through the strand displacement reaction presented in Figure 1C. Starting from the left, a crosslink formed by a pivot‐exchange duplex A/B_1_ leaves accessible the toehold domain β_2_, while the domains α/α^⁎^ and β_1_/β_1_^⁎^ are hybridized. The strand displacement reaction starts when an exchange strand B_2_ hybridize to the free toehold β_2_ to invade the duplex A/B_1_. In the middle panel, B_2_ progressively replaces B_1_ via branch migration. On the right, B_2_ has replaced B_1_ at the α domain, the toehold domain β_1_/β_1_^⁎^ melts, and B_1_ is released. This strand displacement reaction is fully reversible: starting from the right panel, strand B_1_ can invade the duplex A/B_2_ to replace and release B_2_. The toehold domains β_1_ and β_2_ are short enough (6 bases) to spontaneously hybridize and melt at room temperature, allowing the exchange strands B_1_ and B_2_ to switch from one crosslink to another.

**TABLE 1:** Name, repeat sequences and functional domains of the RCA products.

RCA product	Repeat sequence 5’→3’ ^a^	Functional domains
Pivot strand A	**[CACCGA GCACAGCGTCGAGG AGCACC A_30_]_10‐100_**	**[β_1_ α β_2_ A_30_]_10‐100_**
Exchange strand B_1_	**[CCTCGACGCTGTGC TCGGTG ACCTATACGT A_30_]_10‐100_**	[α^*^ β_1_^*^ δ_1_ A_30_]_10‐100_
Exchange strand B_2_	**[TGTTAGTAGT GGTGCT CCTCGACGCTGTGC A_30_]_10‐100_**	[δ_2_ β_2_^*^ α^*^ A_30_]_10‐100_

^a^ We use a space to separate and identify each functional domain in the repeat sequence.

The sequences are designed so that the free energy of both duplexes A/B_1_ and A/B_2_ are equal (*ΔG_A/B1_*  = * ΔG_A/B2 _* =  *‐ΔG_S0→S1 _* =  −119 kJ/mol at 25°C, Table S1) [55]. There is therefore no energetic preference for A to bind to B_1_ or B_2_, which prevents the exchange from being terminated due to saturation with a preferred exchange strand. This is an associative reorganization mechanism, since crosslink connectivity increases during the reconfiguration process. In contrast, reorganization driven by duplex melting is dissociative, as the crosslink connectivity decreases during the reconfiguration process.

**FIGURE 2:**
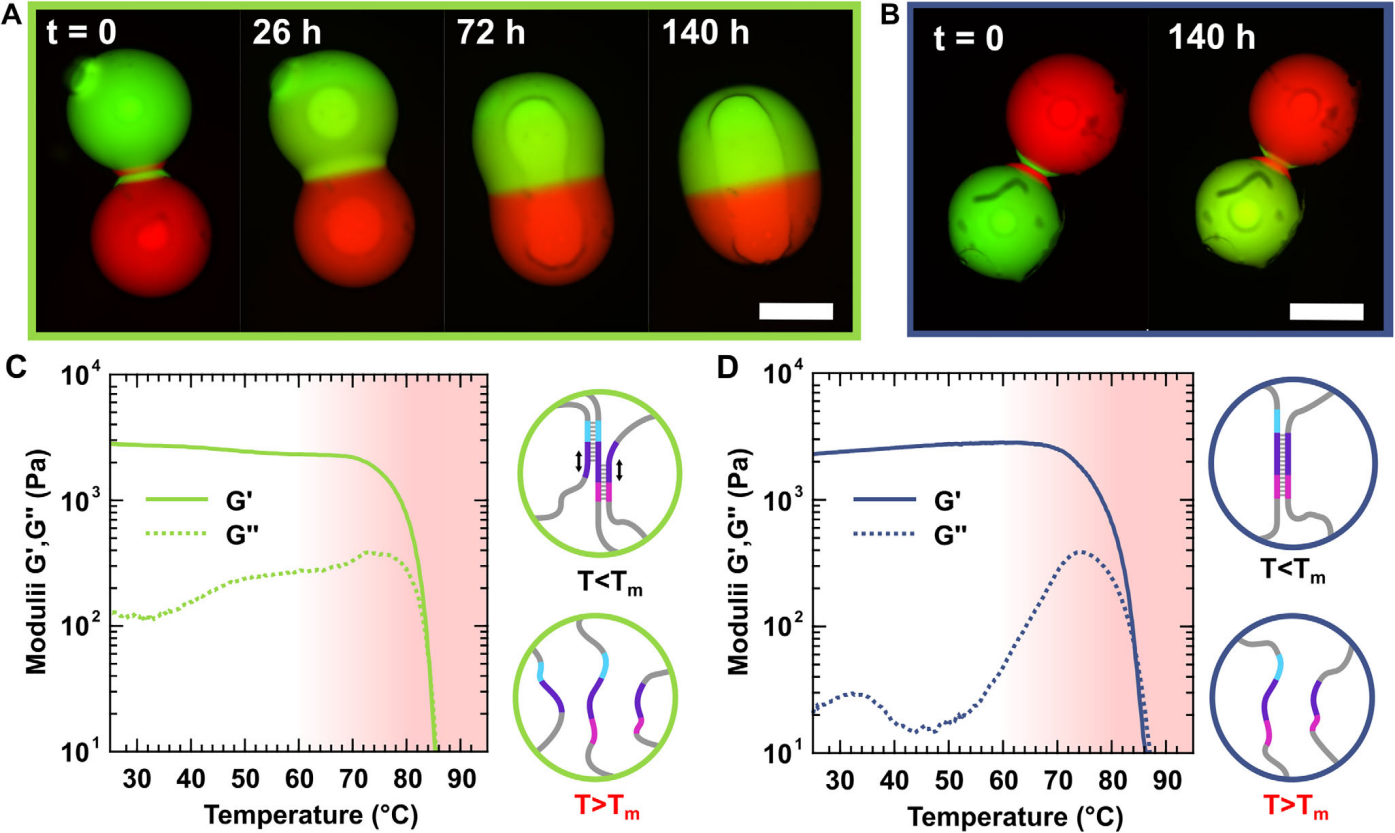
Key features of associative and dissociative DNA hydrogels. (A) Wide‐field microscopy images showing associative DNA hydrogels melding at 30°C. (B) Under the same conditions, dissociative hydrogels cannot reorganize and meld. The hydrogels are immersed in mineral oil to force contact via capillary forces and to prevent water evaporation. Here the DNA strands are fluorescently labeled during synthesis by replacing some of the thymine bases with Atto_488_‐ or Atto_565_‐modified bases (See Note S1). Scale bar = 0.5 mm. (C,D) Evolution of the storage and loss moduli with temperature for associative (C) and dissociative (D) DNA hydrogels. The schemes to the right of each graph represent the predominant crosslinking state below and above *T_m_*, respectively. All hydrogels are composed of the same DNA strands and differ only in the relative proportion of strands A and B. Associative hydrogels contain a two‐fold excess of strand B compared to strand A, whereas dissociative hydrogels have a slight deficit of strand B relative to A. Oscillatory rheology data are collected on 2 wt.% DNA hydrogels at 1 rad·s^−^^1^ and 5% strain.

The nature of the reorganization mechanism, associative or dissociative, significantly influences the macroscopic behavior of DNA hydrogels at room temperature. The characteristic behaviors of DNA hydrogels with and without associative crosslink exchange are presented in Figure 2. The associative crosslink exchange is catalyzed by the presence of free toeholds β_1_^⁎^ or β_2_^⁎^ : Therefore it requires an excess of sequences B_1_ and B_2_ compared to sequence A in order to proceed. To facilitate comparison, all DNA hydrogels presented in this work are made from the same RCA products A, B_1_, and B_2_. The sequences B_1_ and B_2_ play symmetrical roles and are always present in equal amounts. For simplicity, we use B to refer collectively to sequence B_1_ and B_2_ (i.e., [B] = [B_1_] + [B_2_]) and β^*^ to refer collectively to β_1_^*^and β_2_^*^. The only variable controlling the reorganization mechanism is the ratio of sequence A to B. When B sequences are in excess, the hydrogels are associative, because they can reorganize via strand displacement reactions. If A strands are in excess, there are no free toeholds β^*^ available to initiate a strand displacement reaction, and the hydrogel is dissociative. This compositional difference strongly impacts hydrogel dynamics at room temperature. Associative hydrogels can meld at 30°C (Figure 2A), whereas dissociative hydrogels do not (Figure 2B) : the associative crosslink exchange increases the reorganization rate of DNA networks and enables macroscopic reshaping at room temperature.

Associative and dissociative DNA hydrogels also display distinct viscoelastic signatures. We systematically measure the loss and storage shear moduli of associative and dissociative hydrogels as a function of temperature *T* (Figure 2C,D). In these oscillatory shear experiments, the storage and loss moduli represent the elastic and viscous contributions to the material response, respectively. At low *T*, associative hydrogels are more dissipative than dissociative ones: they exhibit a tenfold higher loss modulus at room temperature. In contrast, because both hydrogels have similar network architectures, their storage moduli are comparable. For associative hydrogels, the changes in loss and storage moduli are small and gradual up to 75°C (Figure 2C). We observe a slight increase in the loss modulus and a decrease in the storage modulus, which results in an increase in the exchange rate with temperature. The small amplitude of these variations demonstrates that such hydrogels are remarkably stable over a broad temperature range, while showing significant reorganization even at low temperatures, as demonstrated by their ability to meld (Figure 2A). Above 75°C, both the storage and loss moduli drop rapidly. This marks the melting transition of the crosslinks, a dissociative process that progressively replaces the associative crosslink at high *T*: the DNA duplexes (i.e., crosslinks) shift from a closed to an open state, resulting in loss of network integrity.

For dissociative hydrogels, both the loss and storage moduli remain nearly constant up to 60°C. At these temperatures, the crosslinks are stable but not dynamic, the hydrogel does not reorganize within a reasonable timescale, as shown by the absence of melding (Figure 2B). Between 60°C and 75°C, we observe a sharp increase in the loss modulus: Thermal energy enables dissociative crosslink reorganization, increasing the viscous response of the sample. The proportion of dissociated crosslinks increases with temperature. Below ∼75°C, this proportion is small enough for its effect on the hydrogel's elastic response to remain negligible. However, as for associative hydrogels, above 75°C, the network loses its structural integrity as the duplexes melt. In dissociative reorganization, the temperature range in which dynamic behavior and structural stability coexist is relatively narrow. For example, in dissociative hydrogels, the fraction of dissipated energy, expressed as *tan(δ)* = *G″/G′*, decreases from approximately 0.25 at 75°C to 0.05 at 65°C. In comparison, the associative hydrogels show a similar *tan(δ)*∼0.25 at 75°C, which, however, reaches 0.05 only at 25°C. Thus, the presence of an associative crosslink exchange mechanism increases by a factor of five the temperature range over which a solid hydrogel exhibits significant dissipation. This is a key signature of the associative exchange mechanism, which enables decoupling of reorganization dynamics at room temperature from the thermal stability of the crosslinks.

## 3 Controlling the Reorganization Dynamics using Strand Displacement Reaction

**FIGURE 3:**
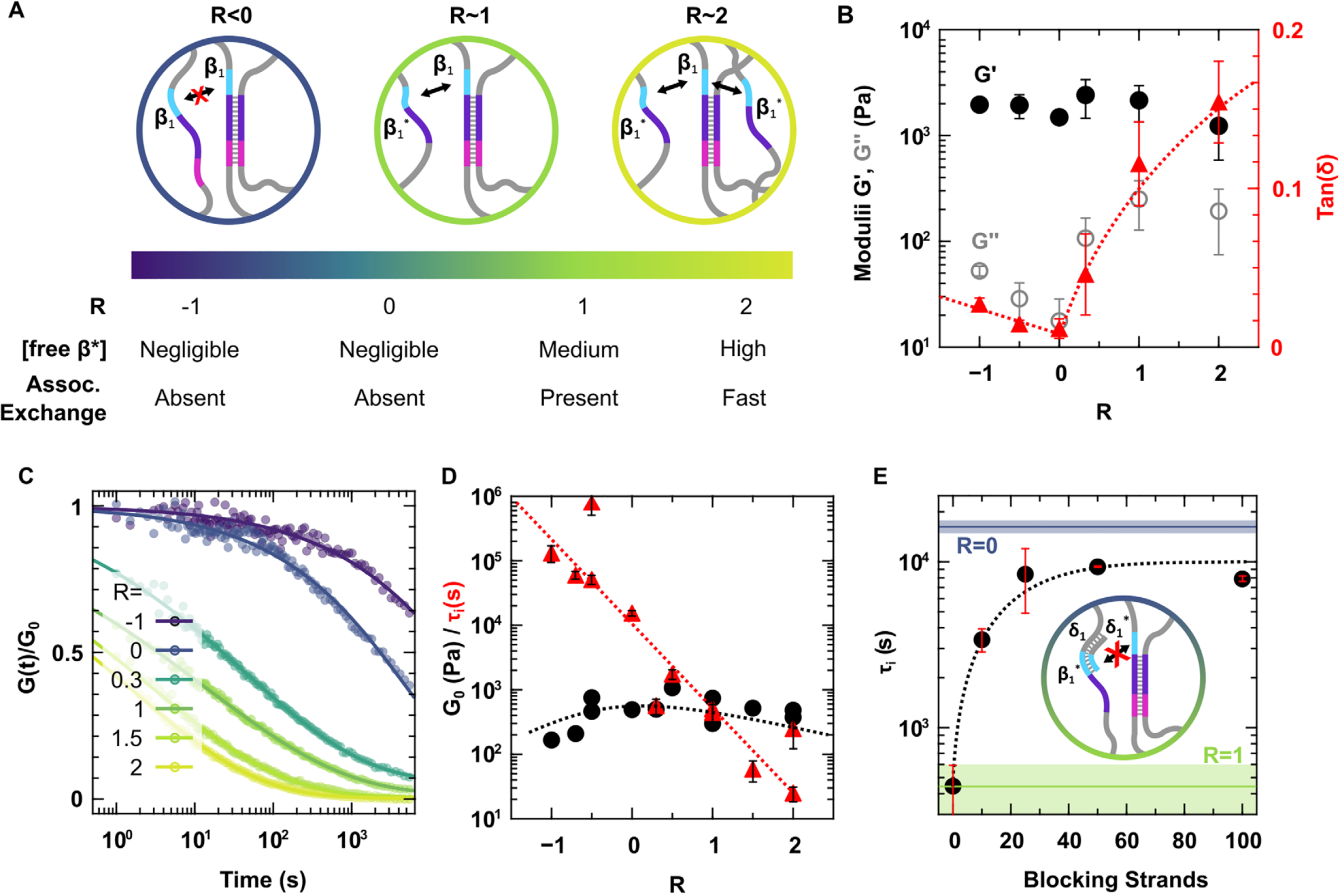
Controlling the reorganization dynamics with associative crosslink exchange. (A) Schematic representation of the effect of sample composition in strands A and B on the availability of catalytic toeholds β^*^ and the resulting reorganization mechanism. (B) Evolution of the elastic (G′, closed black circles) and viscous (*G″*, open gray circles) moduli, and of the loss factor *tan(δ)* (red) of DNA hydrogels as a function of the stoichiometric ratio *R*, as defined in Equation (1). Data were acquired on 2 wt.% hydrogels at 37°C, 1 rad·s^−^^1^, and 5% deformation. Error bars represent the standard deviation across two to five different samples. (C) Stress relaxation experiments for hydrogels with different *R* (all data: 5% strain, 37°C). Symbols represent experimental stress measurements and solid lines are stretched exponential fits (see Equation (2)). (D) Initial relaxation modulus *G_0_* (black circles) and integral relaxation time τ_i_ (red triangles) obtained by fitting the stress relaxation data using a stretched exponential decay. Error bars on *G_0_* are too small to be visible. (E) Effect of adding blocking strands that sequester toeholds (see inset) on the integral relaxation time of an associative hydrogel (*R* = 1). For comparison, green and blue lines represent the integral relaxation times of *R* = 1 and *R* = 0 hydrogels, respectively, without blocking strands; the shaded areas indicate the corresponding error margins. In panels B, D, and E, dotted lines are included as guides to the eye.

The main control parameter of the associative exchange mechanism is the concentration of active catalytic toeholds β^*^. As discussed earlier, the strand displacement reaction requires a free exchange strand to proceed. To compare samples of different compositions, we define the stoichiometric ratio *R*, which gives the proportion of active toeholds relative to the number of crosslinks (Equation (1)):

(1) $$R=\frac{\left[B\right]-\left[A\right]}{\left[X_{AB}\right]}\;$$

where [A] and [B] are the total concentrations of the repeat sequences of strands A and B, respectively, and [X_AB_] is the total concentration of crosslinks (either A/B_1_ or A/B_2_). Since the formation of a crosslink requires one α and one α^*^ domain, the concentration of crosslinks is given by [X_AB_] = min([A], [B]). The scheme in Figure 3A summarizes the effect of the ratio *R* on the associative exchange mechanism. For *R* ≤ 0, all toeholds β^*^ on the B strands are involved in A/B duplexes, so no active toeholds remain to initiate strand displacement. Note that the excess of toehold domains on A strands cannot hybridize to A/B crosslinks to initiate strand displacement. Therefore, for *R* ≤ 0, crosslinks cannot reorganize via an associative exchange mechanism. In contrast, for *R* > 0, the hydrogel contains an excess of exchange strands, meaning that active toeholds β^*^ are available for the associative exchange to occur. By design, *R* is directly proportional to the number of available toeholds capable of initiating strand displacement; therefore, the reorganization rate via associative exchange scales with *R*.

We systematically measured the rheological behavior of DNA hydrogels as a function of their composition *R*. First, we examined the storage (G′) and loss (G″) moduli of the hydrogels in the linear regime (Figure 3B). The loss modulus increases with *R*, i.e., as strand displacement becomes more prevalent, while the storage modulus remains relatively constant. This effect is more clearly observed in the loss factor, *tan(δ)*, which characterizes the relative contribution of dissipation to the mechanical response of the sample. For *R* ≤ 0, *tan(δ)* is low and nearly constant. As expected, it steadily increases with *R* for *R* > 0. These measurements confirm that the proposed associative reorganization mechanism is stoichiometry‐controlled. Note that here the increase of relaxation, either at fixed temperature and increasing R (Figure 3B) or at fixed R and increasing temperature (Figure 2C), causes a decrease of storage modulus (G’). It may seem surprising as associative reorganization has been reported in the literature to increase rigidity due to loop healing or entropic elasticity [56, 57]. However, in our experiments the network reorganization is fast enough even at 37°C such that relaxation hides these effects. To quantitatively assess the timescale of reorganization, we performed stress relaxation experiments (Figure 3C). We fitted the results with a stretched exponential decay, as defined in Equation (2).

(2) $$\sigma \;\left(t\right)=\gamma G_{0}\;e^{-{\left(\frac{t}{\tau_{r}}\right)}^{\beta }}+s$$

where *G_0_* is the initial shear modulus, *γ* the imposed step strain, *τ_r_* the 1/e relaxation time, and *β* the stretching exponent. s is a small baseline correction applied to dynamic samples only, to avoid fitting artifacts arising from experimental uncertainty near the *σ* ≈ 0 baseline. A simple exponential decay (*β*  =  1) corresponds to a single relaxation time, but the stretching exponents measured in our samples (0.2 ≤* β *≤ 0.5) indicate a broad distribution of relaxation times. This distribution is better captured by the integral relaxation time (*τ_i_*) rather than *τ_r_*, although both show similar trends (see also Note S4 and Figure S4 for details). In Figure 3D, we report both τ_i_ and the initial relaxation modulus (*G_0_*) as a function of *R*. Consistent with the oscillatory shear data, the initial relaxation modulus *G_0_* remains relatively constant across compositions, while the relaxation time decreases from *τ_i_ *> 10^5^ s for dissociative hydrogels (*R* ≤ 0) to *τ_i_* < 200 s for associative hydrogels (*R* > 1). Thus, the catalytic control of the associative exchange mechanism provides a convenient means of tuning relaxation times across more than three orders of magnitude. The broad distribution of relaxation times may seem surprising, as dissociative networks typically exhibit narrower distributions. However, the network consists of long DNA strands with tenths of sticky domains along the chain (see Table 1) and comprises entanglements on top of the supramolecular crosslinks. Thus, the complete relaxation requires disentanglement via sticky reptation processes, which involves the sequential breaking and reforming of multiple crosslinking points [58, 59]. The presence of associative crosslink exchange, which prevents some of the topological relaxation permitted in dissociative networks, further amplifies this effect. If needed, such structural contributions can be mitigated by modifying the network topology or by decreasing the length of the RCA products [29].

We have shown the possibility of controlling the reorganization of DNA hydrogels by changing their composition. Importantly, one can also control the reorganization mechanism of an already formed sample. The control of strand displacement reactions using short oligomers that hide or reveal toeholds is well established [46]. Here, we leverage this approach by designing “blocking strands” that hybridize to the toehold and to an adjacent addressing domain δ (either δ_1_ for B_1_ or δ_2_ for B_2_), thereby hiding the toehold β^*^ and suppressing associative exchange. The addressing domain is necessary to stabilize the hybridization of the blocking strands to the network. Indeed, as mentioned earlier, without stabilization, a six‐base‐long strands complementary to the toeholds would continuously melt and rehybridize at room temperature, and hence would not block the associative exchange efficiently. As shown in Figure 3E, adding 10 mol% of blocking strands (relative to the total number of β^*^ toeholds) increases the relaxation time of an *R* = 1 hydrogel from 200 to 2000 *s*. Further increasing the concentration of blocking strands to 25%–50% of toehold concentration increases the relaxation time up to 9000 *s*, comparable to that of a dissociative hydrogel with *R* = 0. Note that we expect no additional effect from increasing the concentration of blocking strands beyond 50% of toeholds. Indeed, for *R* = 1, 50% of the toeholds β^*^ are already engaged in crosslinks and thus inactive; therefore, one expects 50% of blocking strands to be sufficient to block 100% of the remaining catalytically active toeholds. Interestingly, we observe a plateau in relaxation time at 25%, earlier than expected, possibly because blocking a sufficiently large fraction of the available toeholds already introduces enough constraints in the relaxation process to significantly slow it. While the origin of this premature saturation remains to be fully clarified, these experiments demonstrate that blocking strands are highly effective in controlling in situ the reorganization speed of already formed dynamic hydrogels.

## 4 Thermodynamic Understanding of the Reorganization Mechanism

**FIGURE 4:**
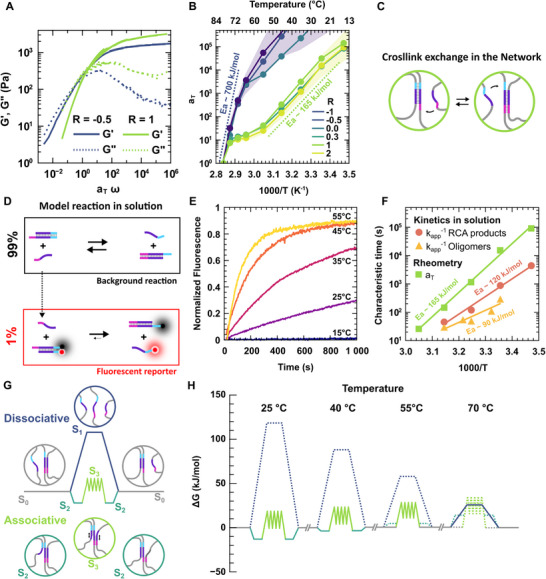
Rationalizing the temperature dependence of associative and dissociative exchange mechanisms. (A) Rheological master curves of dissociative (*R* = −0.5) and associative (*R* = 1) DNA hydrogels obtained by time‐temperature superposition of frequency sweeps performed between 15°C and 85°C, using the shift factors *a_T_* shown in (B). The reference temperature is 85°C, where both hydrogels reorganize via dissociative mechanisms and exhibit similar dynamics. (B) Arrhenius plot showing the evolution of the shift factor *a_T_* with *1000/T* for DNA hydrogels with different compositions. The shaded areas in blue and green indicate the error intervals for *R* = −0.5 and *R* = 1, respectively (see Figure S5 for details). The slopes of the curves are proportional to the activation energy of the dominant reorganization mechanism at a given temperature. (C) Schematic representation of strand displacement reaction driving the associative crosslink exchange in the network. (D) Schematic representation of the model strand displacement reaction used to monitor the kinetics of exchange in solution. (E) Evolution of fluorescence with time after adding the fluorescent reporter at *t* = 0, measured at different of temperatures for a constant stoichiometric ratio *R* = 1 for the background reaction. (F) Evolution of the characteristic time of associative exchange with *1000/T*, measured as *k_app_^−1^* for model reactions using short oligomers and long RCA products in solution (yellow triangles and red circles, respectively), plotted alongside the corresponding *a_T_* values obtained by TTS for macroscopic hydrogels (green squares). See Note S5 and Figure S6 for details. (G) Schematic representation of the free energy profiles for dissociative (top, blue) and associative (bottom, green) crosslink exchange mechanisms. The curves represent the evolution of the free energy (*ΔG*) of intermediate conformations for both mechanisms. The S_3_ state is shown as a sawtooth profile, since during branch migration, each nucleobase step is associated with a variation in *ΔG* [61]. (H) Quantitative evolution of the energetic profiles of exchange with temperature (see Notes S6 and S7 and Figure S7 for details). The solid lines represent the most favorable pathway (associative or dissociative) that minimizes *ΔG* along the path. The dotted lines indicate the alternative, less favorable pathways, shown for comparison.

We expect the reorganization speed of dynamic hydrogels to increase with temperature, as higher temperatures generally accelerate chemical reaction rates. Investigating how reorganization dynamics vary with temperature can further offer valuable insights into the underlying thermodynamic landscape governing the crosslink exchange reactions. We performed frequency sweeps on DNA hydrogels with stoichiometric ratios varying from *R* = −1 to *R* = 2, at multiple temperatures ranging from 15°C to 85°C. We constructed master curves for *G′* and *G″* by applying the Time–Temperature Superposition (TTS) principle (details in Note S5) [60]. The characteristic master curves for associative (*R* = 1) and dissociative (*R* = ‐0.5) hydrogels are shown in Figure 4A. Overall, both master curves exhibit Maxwell‐like features, including terminal relaxation at low frequency, a maximum of *G″* near the *G′/G″* crossover, and a plateau at high frequencies. However, the overall shape of the master curves deviates from that of an ideal Maxwell material, as both dynamic and static hydrogels reach the peak of dissipation at frequencies higher than the *G′/G″* crossover. This is typical of materials with a broad distribution of relaxation times, as previously identified in stress relaxation experiments (Figure 3C,D) [59]. The dissociative hydrogels display a well‐defined dissipation peak with a single maximum, suggesting that a single chemical process governs relaxation. In contrast, the dissipation peak of associative hydrogels exhibits a shoulder, suggesting the presence of multiple relaxation mechanisms [22]. We attribute this feature to the transition from associative exchange at low temperatures to dissociative exchange at high temperatures.

The shift factors *a_T_* obtained from TTS reflect the underlying reorganization mechanism. The values of *a_T_* for hydrogels with stoichiometric ratios from *R* = −1 to *R* = 2 are presented in Figure 4B. Strikingly, the evolution of *a_T_* with temperature depends only on the type of reorganization mechanism, not on the exact value of *R*. Hydrogels with associative mechanisms (*R* > 0) collapse onto a single *a_T_* curve, while dissociative hydrogels (*R* ≤ 0) collapse onto a different curve. For materials that reorganize via reaction‐controlled processes, such as DNA melting or strand displacement, *a_T_* is proportional to the reaction rate constant *k*. Therefore, *a_T_* follows an Arrhenius dependence on temperature, as described in Equation (3):

(3) $$a_{T}\propto k=A\cdot e^{\left(-\frac{E_{a}}{R_{g}\cdot T}\right)}$$

where *E_a_* is the activation energy of the reaction, *R_g_* the ideal gas constant, *T* the absolute temperature, and *A* the Arrhenius prefactor of the reaction. By plotting *a_t_* as a function of *1000/T* (i.e., in an Arrhenius plot), we can identify the temperature ranges where the reorganization is dominated by a single chemical process (Figure 4B). At low temperatures, between 15°C and 55°C, all associative hydrogels (*R* > 0) exhibit similar slopes, corresponding to an activation energy of 165 ± 20 kJ/mol. We attribute this energy barrier to the rate‐limiting step of the strand displacement reactions. Dissociative hydrogels, on the other hand, display a significantly higher activation energy in the range of ∼700 kJ/mol, only measurable between 50°C and 85°C. At lower temperatures, the evolution of the modulus with *T* is too small to allow reliable measurement (the error on *a_T_* becomes very large), while at higher temperatures the network loses its structural integrity and the moduli become too low to measure. Interestingly, we find that associative hydrogels show a similar slope to dissociative ones between 75°C and 85°C, indicating that, as expected, the dissociative pathway dominates reorganization at high temperatures. Surprisingly, we also observe a flattening of the Arrhenius slope between 60°C and 75°C, suggesting a shift in the rate‐limiting step controlling the associative crosslink exchange. The activation energies of such supramolecular associative or dissociative crosslink exchange may appear surprisingly high, in comparison to covalent vitrimers, that have activation energies in the range of 40 to 140 kJ/mol [35]. However, DNA hydrogels generally display high activation energies, e.g., as previously reported in the case of dissociative star‐shaped DNA hydrogels [28]. This high activation energy emerges from the unique cooperative base stacking of DNA, that gives DNA duplexes high enthalpies of formation. One can, in principle, calculate these thermodynamic parameters in silico to rationalize reorganization processes (Note S6) [62]. Yet, it is necessary to isolate network contributions from individual crosslink kinetics before discussing in more detail the thermodynamics of the associative crosslink exchange [63].

In order to decorrelate network effects from strand displacement kinetics, we designed a model reaction, inspired by scrambling experiments in vitrimers, that mimics in solution the associative crosslink exchange [37]. The model reaction, schematized in Figure 4D, consists of two parts. First, a background reaction mimics the crosslink exchange mechanism of the network, but in solution. This reaction can rely on commercial oligomers that have the same sequence as the sticky parts of the network (β_1_αβ_2_, α^*^β_1_^*^ and β_2_^*^α^*^) or on diluted RCA products that do not form a percolating network. Second, we introduce a reporting construct, which consists of a duplex (β_1_αβ_2_/α^*^β_1_^*^) functionalized with a fluorophore (Atto_565_) on one side and with a quencher (QXL_570_) on the other side. Such construct is initially non‐fluorescent due to Fröster Resonance Energy Tranfert between the fluorophore and the quencher. When added in small quantity into the background solution, the exchange of the quencher functionalized strand by an unlabeled strand results in fluorescence increase. The evolution of the fluorescence with time at different temperatures for a R = 1 mixture is presented Figure 4E, and can be fitted using first‐order kinetics (Figure S6). The evolution of the reaction constant with temperature both in the case of RCA products or short commercial oligomers is presented in an Arrenhius plot, Figure 4F. The activation energy in solution goes from 90 ± 15 kJ/mol for short oligomers up to 120 ± 10 kJ/mol for diluted RCA products. Hence, the presence of dangling ends at the extremities of the invading strand increases the activation energy of the exchange. This effect is even more pronounced when these dangling ends are immobilized in a percolating 3D network as measured in rheology.

In order to go one step further, we propose to rationalize this behavior using molecular thermodynamics. The thermodynamic parameters controlling DNA hybridization and strand displacement reactions are well established. Leveraging the conceptual framework of the energy landscape elucidated by Winfree et al. [61] and UNAFold simulations [55, 64], we present a quantitative diagram of the reaction pathways, in free energy, for the associative and dissociative crosslink exchanges across a range of temperatures (Figures 4G,H, see also Notes S6 and S7 and Table S1 for details). At room temperature, the free energy barrier for dissociative exchange (blue line) is much higher than that of associative exchange (green line). Yet, since the configurational entropy of the dissociated state (S_1_) is higher than that of the associated 3‐strand complex (S_3_), the free energy barrier for the dissociative exchange path decreases more rapidly with temperature than that of the associative exchange path, due to the contribution of the –*TΔS* term in the free energy. The free energy barrier of the two paths become comparable around 60°C. The profiles also predict an intermediate regime, between 45°C and 60°C, where toehold hybridization is unfavorable (*ΔG > 0*), but where the associative exchange still minimizes the free energy barrier. In this regime, illustrated by the diagram at 55°C in Figure 4H, the exchanges proceed via direct invasion of the duplex without a stable S_2_ state. A noteworthy observation is the consistent high temperature shift of the transitions measured in TTS relative to the predictions derived from our thermodynamic profiles. This discrepancy is primarily attributable to the fact that the thermodynamic parameters used to construct the energy landscape are taken from studies on short oligomers in dilute solution, thereby omitting the contributions arising from network effects [61, 65]. Nevertheless, such detailed reaction profiles offer a robust framework for the qualitative identification of reaction pathways and the elucidation of rate‐limiting steps controlling molecular reorganization at different temperatures.

The quantitative rationalization of the activation energy for associative and dissociative exchange, based on thermodynamic profiles, is inherently difficult because identifying unambiguously the limiting step of such multistep processes is difficult (see Note S6). Nonetheless, meaningful insight can be obtained by comparing the thermodynamic parameters for associative crosslink exchange, Table S1, with the activation energies measured in solution using short oligomers. We observe that the activation energies (90 ± 15 kJ/mol) for associative exchange match the enthalpy variation calculated for the initiation of strand invasion (ΔH_S2→S3_ = 88 kJ/mol). Similarly the activation energy for dissociative reorganization (700 kJ/mol) corresponds to the enthalpy variation for duplex melting (ΔH_S0→S1_ = 735 kJ/mol). Hence, these thermodynamic pathways, provide a rationale behind the apparently high activation energies found in associative DNA hydrogels compared to traditional vitrimers. Beyond interpreting their linear viscoelastic response, these thermodynamic considerations also offer a robust framework for elucidating rupture mechanisms operating in the nonlinear regime, as discussed hereafter.

## 5 Decoupling Dynamics and Rupture with Associative Exchange Mechanism

**FIGURE 5:**
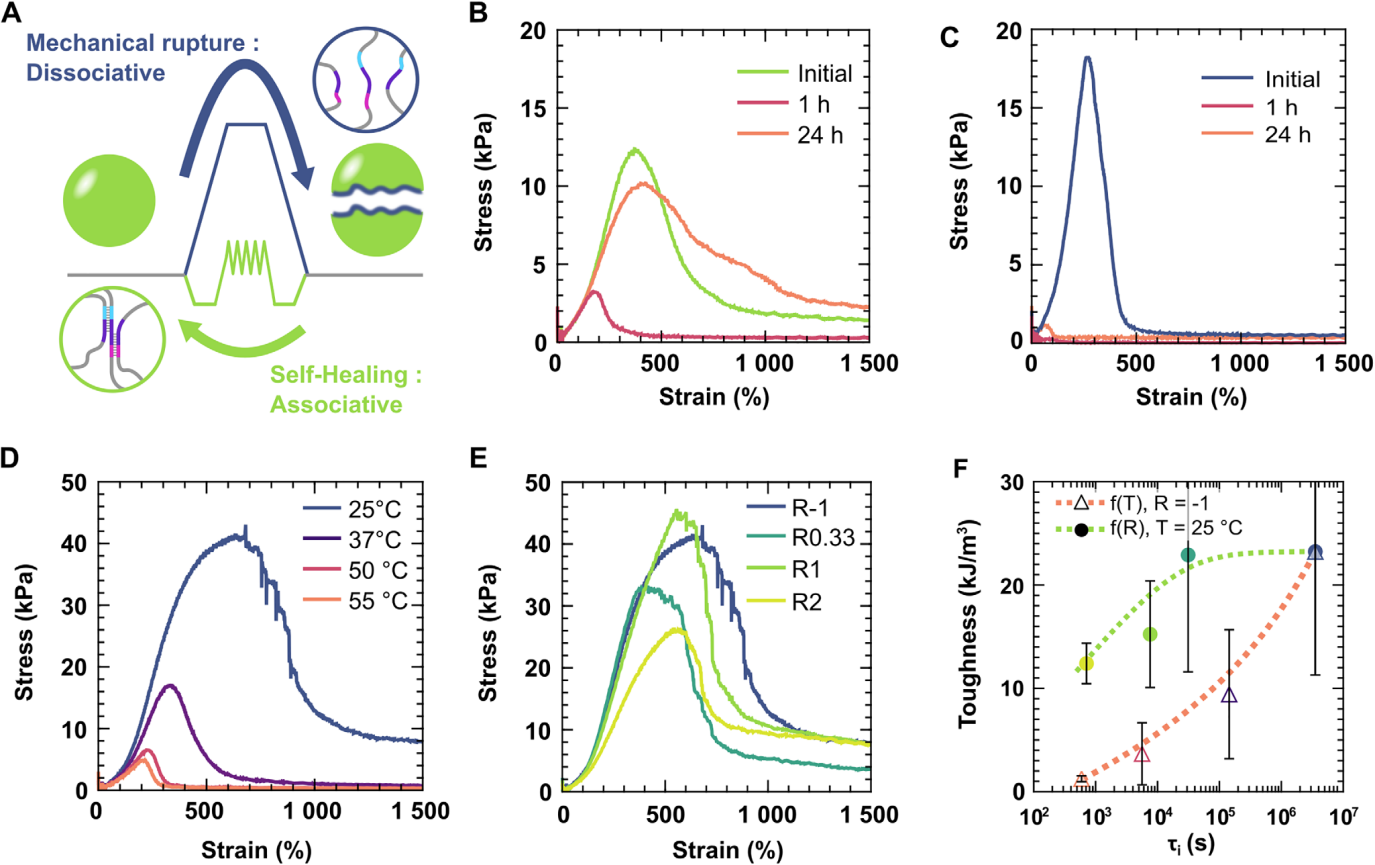
Rupture and self‐healing of associative and dissociative DNA hydrogels. (A) Schematic representation of the two different pathways for rupture and self‐healing of associative DNA hydrogels. Rapid mechanical rupture involves the dissociation of the DNA crosslinks, which has a high free energy barrier at room temperature. In contrast, self‐healing is slower but relies on a strand displacement reaction that has a low free energy barrier. (B,C) Start‐up shear experiments on DNA hydrogels with and without associative exchange, respectively, as prepared (initial), and after 1 h or 1 day at 37°C after rupture. (D) Start‐up shear experiments for dissociative DNA hydrogels performed at various temperatures, for R = −1. The sample weakens with increasing temperature. (E) Start‐up shear experiments for associative DNA hydrogels with increasing stoichiometric ratio *R*. The sample strength is almost independent of *R*. (F) Graph illustrating the evolution of the toughness of associative and dissociative hydrogels as a function of their reorganization dynamics. The toughness is measured as the area under the stress peak between 0% and 1000%. Error bars correspond to the standard deviation from measurements on three different hydrogels. The reorganization speed is quantified by the corresponding integral relaxation time τ_i_. The τ_i_ at different temperatures are calculated by multiplying the τ_i_ measured at 37°C (Figure 3) with by the corresponding *a_T_* measured in TTS (Figure 4).

We have shown that the presence of an associative exchange enables the decoupling of hydrogel dynamics from thermal stability. Here, we take a step further by examining how exchange mechanisms influence the rupture of the network. Ultimately, the rupture strength of a supramolecular hydrogel is determined by the intrinsic strength of its crosslinks, although the overall network architecture also plays a significant role [4]. For dissociative hydrogels, the rupture and relaxation pathways are identical, both involving the opening of crosslinks. In contrast, associative hydrogels offer two possible pathways (Figure 5A). On one hand, the associative crosslink exchange is active at rest under ambient conditions due to its low free energy barrier, but its rate is limited by the concentration of active catalytic domains (free β^*^). This pathway governs the relaxation dynamics of unperturbed samples, as previously discussed. On the other hand, the dissociative pathway is almost inactive at room temperature due to its high free energy barrier. However, dissociation can be mechanically activated under stress [50]. This dissociative pathway governs the hydrogel's rupture strength at high strain rates, since its rate is not limited by the presence of catalytic sites. In startup shear experiments, both pristine dissociative and associative hydrogels exhibit similar rupture strengths (Figure 5B,C), indicating that their rupture proceeds via similar dissociative mechanisms. However, only the associative hydrogel can self‐heal, within 24 h, thanks to the low free energy barrier of its reorganization mechanism. Note that, although dissociative hydrogels can relax some stress at 37°C (see Figure 3C), they are unable to self‐heal because reforming a pristine‐like network at the rupture interface requires timescales that are orders of magnitudes longer than those for the bulk stress relaxation in the linear regime. Thus, the associative exchange mechanism enables self‐healing without compromising the mechanical strength of the material.

While both dissociative and associative reorganization pathways control hydrogel relaxation, they affect toughness differently. In dissociative mechanisms, the hydrogel's relaxation time is dictated by the dissociation rate of crosslinks, which can be modulated by temperature [33]. Increasing temperature lowers the free energy barrier of the dissociative pathway (Figure 4H), thereby accelerating relaxation. In contrast, in associative mechanisms, the characteristic relaxation time is governed by the concentration of catalytically active domains, quantified by the stoichiometric ratio *R* (Figure 3D). To compare how each mechanism affects hydrogel strength, we perform startup shear tests while varying either *T* or *R*, keeping the shear rate constant at 1 s^−^^1^. Figure 5D shows the rupture behavior of dissociative hydrogels (*R* = −1) as temperature increases from 25°C to 75°C. While the slope at the origin remains unchanged, consistent with the nearly constant *G’* over this temperature range (Figure 2D), the rupture strength (i.e., the maximum stress reached during startup shear) decreases rapidly with temperature. This behavior contrasts with that of associative hydrogels tested at constant temperature (T = 25°C) while varying composition (*R* = [−0.5; 2], Figure 5E). In this case, both the slope at the origin and the rupture strength remain relatively constant. Only the fastest‐reorganizing samples (*R* = 2) exhibits a slight decrease in rupture stress, likely due to rapid rearrangement enabling partial stress relaxation prior to failure. To quantify the toughness, defined as the energy stored before rupture, we calculate the area under the stress–strain curves and plot it against the characteristic time of reorganization τ_i_ (Figure 5F3). For dissociative reorganization, hydrogel toughness decreases as reorganization dynamics increase (i.e. as τ_i_ decreases). This is expected, as lowering the free energy barrier of reorganization also lowers the energy barrier for rupture (Note that a similar effect is observed for associative hydrogels, see Figure S8). In contrast, for associative reorganization, toughness is almost unaffected by the reorganization speed. Indeed, increasing the exchange rate via network composition does not affect the energy profile of the rupture pathway, hence, the energy required to break the network remains unchanged. These results highlight a fundamental distinction between associative and dissociative dynamics: While both govern relaxation, only dissociative mechanisms inherently trade off mechanical strength for faster reorganization.

## 6 Conclusion

Our work reveals that associative crosslink exchange allows for the decoupling of network reorganization from thermal and mechanical stability in supramolecular hydrogels. This finding challenges the “strong means slow” paradigm for supramolecular networks, which holds only for dissociative reorganization. By controlling the mechanism of the reorganization, specifically through associative crosslink exchange, it is possible to produce supramolecular networks that differ in reorganization speed by more than three orders of magnitude, yet have similar architecture, melting temperature, and rupture toughness. We provided a comprehensive description of the macroscale rheological behavior of both associative and dissociative DNA hydrogels, as well as a thermodynamic framework to rationalize this behavior. DNA is an ideal platform for this systematic comparison, as it enables the formation of both associative and dissociative hydrogels from the exact same DNA strands, only changing their proportions altered. Beyond DNA‐based materials, these findings reveal a general design principle for supramolecular hydrogels: the reorganization mechanism is a key determinant of dynamic and mechanical properties. Associative crosslink exchange decouples reconfigurability from mechanical integrity, enabling the rational design of adaptive hydrogels that maintain thermal and mechanical stability. This work illustrates how reorganization pathways can be leveraged to control macroscale behavior across a wide range of dynamic materials. By identifying the reorganization mechanism as a key parameter, we aim to stir the development of hydrogels that better mimic the mechanical and adaptive properties of biological tissues, while bridging the gap between self‐healing ability and structural robustness.

## 7 Experimental Section/Methods

### 7.1 Materials

ssDNA oligomers are purchased from Eurogentec, as summarized in Table 2. The enzymes T4 DNA ligase (2.5 WU µL^−^^1^), Exonuclease I (20 U µL^−^^1^), as well as deoxynucleotide triphosphates (dATP, dTTP, dGTP, and dCTP; 100 µM) and fluorescent deoxynucleotide triphosphates (Aminoallyl‐dUTP‐XX‐ATTO‐488 and Aminoallyl‐dUTP‐XX‐ATTO‐594) are purchased from Jena Bioscience. Φ29 DNA polymerase (10 U µL^−^^1^) is obtained from LGC Biosearch, and inorganic pyrophosphatase (0.1 U µL^−^^1^) from Thermo Fisher. Magnesium acetate (MgAc_2_), sodium chloride (NaCl), calcium acetate (CaAc_2_), silicone oil, tris(hydroxymethyl)aminomethane hydrochloride (Tris‐HCl and Trizma buffer, pH 7.5), and poly‐D‐lysine (300 kg mol^−^^1^) are purchased from Sigma–Aldrich. Disodium ethylenediaminetetraacetate dihydrate (EDTA) is obtained from Euromedex. DNA strands are diluted and stored in TE buffer, which contains 10 mm Tris(hydroxymethyl)aminomethane (pH 8.0) and 1 mm EDTA. For hydrogel assembly and characterization, the TE buffer is supplemented with 100 mm NaCl and 10 mm magnesium acetate (MgAc_2_), referred to as TENaMg buffer. DNA strands are stored at −25°C in TE buffer.

**TABLE 2:** Commercial DNA oligomer used, with their name, purification grade, and modifications.

	Name	Sequence 5’→3’	Purification	Modification
Linear Templates for RCA	Tp_A	GCTGTGCTCGGTGTTTTTTTTTTTTTTTTTTTTTTTTTTTTTGGTGCTCCTCGAC	HPLC	5’‐Phosphorylation
	Tp_B_1_	GTCGAGGTTTTTTTTTTTTTTTTTTTTTTTTTTTTTACGTATAGGTCACCGAGCACAGC	HPLC	5’‐Phosphorylation
	Tp_B_2_	GTCGAGGAGCACCACTACTAACATTTTTTTTTTTTTTTTTTTTTTTTTTTTTGCACAGC	HPLC	5’‐Phosphorylation
Ligation strands	Lig_A	GCACAGCGTCGAGG	HPLC	None
	Lig_B_1/2_	CCTCGACGCTGTGC	HPLC	None
RCA Primers	Prim_A	GCACAGCGTCGA*G*G	RP‐Cartridge—Gold	Phosphorothioated at position*
	Prim_B_1/2_	CCTCGACGCTGT*G*C	RP‐Cartridge—Gold	Phosphorothioated at position*
Fluorescent oligomer	Atto_488_	TTTTTTTTTTTTTTTTTTTTT	HPLC	5’ Atto488
Blocking Strands	Block‐B_1_	ACGTATAGGTCACCGA	SePOP Desalted	None
	Block‐B_2_	AGCACCACTACTAACA	SePOP Desalted	None
Molecular kinetics	xA	CACCGAGCACAGCGTCGAGGAGCACC	HPLC	None
	xB_1_	CCTCGACGCTGTGCTCGGTGACCTATACGT	HPLC	None
	xB_2_	TGTTAGTAGTGGTGCTCCTCGACGCTGTGC	HPLC	None
	xA_Q_	CACCGAGCACAGCGTCGAGGAGCACC	HPLC	3’‐QXL_570_
	xB_1F_	CCTCGACGCTGTGCTCGGTGACCTATACGT	HPLC	5’‐Atto565

Thermal ramps (up to 50 µL) are performed on a thermocycler (T100, Bio‐Rad), while larger volumes are heated using a thermo shaker (PMHT, Grant Instruments). ssDNA concentrations are measured using a DeNovix DS‐11 FX+ spectrophotometer, assuming a standard conversion factor of 33 µg·OD_260_^−^^1^. Capillary gel electrophoresis is conducted on a Qsep1 system (BiOptic Inc.), using standard DNA cartridges for short oligomers. Rheological measurements are carried out on an MCR 302 rheometer (Anton Paar) equipped with a sanded cone‐plate geometry (diameter 8 mm, angle 3°, gap 50 µm; part number CP8‐3) and a temperature‐controlled oven (H‐PTD 200). Fluorescence measurements are conducted using a Cary Eclipse fluorimeter (Agilent Technologies) equipped with a Peltier temperature controller. Fluorescence imaging is performed on an inverted microscope (Leica DMi8) using a 10× air objective (NA  =  0.25), a LED excitation lamp (CoolLED pE‐300), and a CMOS camera (C13440 ORCA Flash 4.0, Hamamatsu).

### 7.2 DNA Synthesis

The DNA synthesis follows three main steps: the circularization of linear templates, the removal of unligated products, and template amplification via rolling circle amplification (RCA). A schematic representation of the DNA synthesis process, and the corresponding electropherogram are provided in Figure S1. For circularization, 4 µL of 10× T4 DNA Ligase buffer (Jena Bioscience; 500 mm Tris‐HCl, pH 7.8 at 25°C; 100 mm MgCl_2_; 100 mm DTT; 10 mm ATP; 25 mg mL^−^^1^ BSA) is diluted in 32 µL of ultrapure water. Then, 2 µL of linear templates (100 µm in TE) and 2 µL of ligation strands (100 µm in TE) are added. The mixture is heated to 85°C, then cooled to 25°C at 0.5°C min^−^^1^. After cooling, 2 µL of T4 DNA Ligase (2.5 WU µL^−^^1^) is added, and the mixture is incubated for 3 h at room temperature. The enzyme is inactivated by heating to 70°C for 20 min. Unligated products are removed using Exonuclease I (3 µL, 20 U µL^−^^1^) and Exonuclease III (1 µL, 200 U µL^−^^1^), which digest linear DNA. Circular templates are protected due to the absence of free ends. The mixture is incubated overnight at 37°C, then heated to 80°C for 40 min to inactivate the enzymes. Templates are purified using Amicon Ultra centrifugal filters (10 kDa cutoff, Merck Millipore) and rinsed three times with TE buffer. ssDNA concentrations are measured with a DS‐11 FX+ spectrophotometer (DeNovix) and diluted to 1 µm. Capillary gel electrophoresis confirms circularization, as circular templates migrate faster than linear ones (Figure S1C). To verify enzyme activity, the same protocol is followed but T4 Ligase is replaced with water. If the final DNA concentration falls below the spectrophotometer's detection limit, exonucleases are active and digest unligated DNA.

For RCA, 100 µL of 10× Φ29 DNA Polymerase buffer (Lucigen; 500 mM Tris‐HCl, 100 mm (NH_4_)_2_SO_4_, 40 mm DTT, 100 mm MgCl_2_) is mixed with 770 µL ultrapure water, 50 µL circular templates (1 µm), 6 µL primer (10 µm), 20 µL Φ29 DNA Polymerase (10 U µL^−^^1^), and 1 µL pyrophosphatase (2 U µL^−^^1^). The mixture is incubated for 30 min at room temperature. Then, 10 µL of a custom dNTP mix (100 µm total, base ratios matched to product sequence) is added to initiate amplification. The reaction proceeds for 48 h at 30°C, followed by enzyme inactivation at 80°C for 10 min. The product is concentrated using Amicon Ultra filters (30 kDa cutoff) and rinsed three times with 400 µL TE buffer. It is then rediluted in 200 µL TE, homogenized at 95°C for 10 min, and quantified using the DS‐11 FX+ spectrophotometer, assuming 33 µg OD_260_^−^^1^ for ssDNA. The RCA stock solution is adjusted to 1 g L^−^^1^ in TE and stored at −20°C. Capillary gel electrophoresis with standard S2 cartridges confirms the formation of long products (>5000 bp), though they cannot be accurately quantified (Figure S1B).

### 7.3 Hydrogel Assembly

A schematic representation of the hydrogel formation process, as well as relevant confocal fluorescence microscopy images, are provided in Figure S2. The hydrogel assembly process relies on liquid–liquid phase separation in the presence of divalent cations, here, calcium, to form dense all‐DNA microgels [66]. The microgels are then washed to remove the divalent cations that induce phase separation and are subsequently concentrated by centrifugation. Above a DNA concentration of 10 g L^−^^1^, the microgels form a homogeneous hydrogel upon thermal treatment. In practice, RCA products (A, B_1_, B_2_), initially at 1 g L^−^^1^, are mixed in the desired proportions in TE buffer (10 mm Tris, pH 7.5, 1 mm EDTA) to reach a final concentration of 0.5 g L^−^^1^, typically in a 250 µL volume. The mixture is heated to 95°C for 5 min in a thermal shaker with vigorous stirring for homogenization, then cooled at 4°C for 5 min (Figure S2B). Once cooled, calcium acetate (0.5 m in ultrapure water) is added to reach a final Ca^2^^+^ concentration of 50 mm. The solution is then heated again to 95°C for 5 min, without agitation, to induce phase separation and form DNA microgels. After cooling to room temperature, the microgels are washed with TENaMg buffer (10 mm Tris, 1 mm EDTA, 100 mm NaCl, 10 mm MgAc_2_) through three cycles of centrifugation (5000 g, 3 min) and redispersion in fresh buffer. The DNA concentration in the supernatant remains below 10 mg L^−^^1^, confirming efficient separation. Although the TENaMg buffer does not induce phase separation at elevated temperatures, the DNA microgels remain stable due to supramolecular crosslinks between α/α^*^ domains. Before the final centrifugation, 2 µL of the suspension is diluted in 18 µL of TE buffer and heated at 95°C for 5 min to measure the DNA concentration. The microgel suspension is concentrated by removing the supernatant until the target DNA concentration (2 wt.%) is reached. The final suspension remains fluid and can be pipetted easily, for example, to load into a rheometer. Finally, the hydrogel is formed in situ by heating the suspension to 95°C for 5 min, dissolving the microgel particles (due to the absence of calcium), and cooling to room temperature to form a homogeneous 3D network.

Pristine RCA products are difficult to handle due to their high viscosity even in the absence of crosslink. The treatment at 95°C in TE (10 min in the synthesis and 5 min in hydrogel assembly) help to reduce the size of the RCA products which is necessary to facilitate handling. We show in Figure S3, the effect of the thermal treatment on the length of the RCA product and on their resulting mechanical behavior of the DNA hydrogels.

### 7.4 Rheology Measurements

The inertia of the rheometer, with and without geometry, is recalibrated in air before each experiment. The surfaces of the rheometer in contact with the sample are thoroughly cleaned using ethanol and ultrapure water. To avoid slipping at high shear stress, the surfaces are coated with poly‐D‐lysine (300 kg mol^−^^1^) by loading in the rheometer 10 µL of poly‐D‐lysine solution at 1 g/L in ultrapure water, letting it adsorb for 5 min, rinsing three times with 20 µL of ultrapure water, and drying with compressed air. This protocol prevents interfacial slipping at high stress without affecting the bulk mechanical behavior of the hydrogels in the linear regime. We show in Figure S8 the effects of the surface treatment on the hydrogel's adhesion to the rheometer geometry and on startup shear measurements.

After surface treatment, the hydrogel is formed directly in the rheometer gap. About 10 µL of microgel suspension at the target concentration (2 wt.%) is loaded directly onto the bottom plate of the rheometer and the target gap is imposed. Once the desired gap is reached (50 µm), any excess suspension is removed (less than 2 µL), and a few milliliters of low‐viscosity silicone oil (47V100 from VWR, viscosity 100 cSt) are deposited at the sample rim to prevent evaporation. Finally, the oven (H‐PTD200) is placed around the sample to ensure homogeneous heating of the sample. The hydrogel is formed in situ by heating the sample to 95°C for 5 min and cooling it down to 25°C at a rate of 0.05°C/s. This heating step melts all supramolecular crosslinks and reforms the 3D network in a well‐defined, reproducible state.

Measurements of storage and loss moduli (e.g., during temperature ramps) are performed in the linear regime by imposing oscillatory shear at 5% strain and a frequency ω  =  1 rad·s^−^^1^. Frequency sweeps are performed at 5% strain for ω in the range of 0.1 to 10 rad·s^−^^1^. Amplitude sweeps are conducted at ω  =  1 rad·s^−^^1^ and between 0.1% and 1000% applied strain. Stress relaxation experiments are performed with a 5% step strain, recording the stress every 0.5 s for 7000 s. When imposing a temperature change, all ramps are conducted at 0.05°C/s. Once the sample reaches the correct temperature, it is left to equilibrate for at least 5 min before measurements begin. Most experiments are conducted at 37°C for two reasons: First, because it is a biologically relevant temperature for human‐related research; second, because it accelerates reorganization and shortens the time required to measure relaxation and self‐healing. Experiments conducted at 25°C (room temperature) show similar behavior but exhibit slower relaxation dynamics.

### 7.5 Time Temperature Superposition

For time temperature superposition tests, the hydrogel is first melted at 85°C, before performing frequency sweeps at 85, 80, 75, 70, 65, 55, 45, 35, 25, and 15°C. At each temperature, the sample is equilibrated for 5 min before starting the test. The storage and loss moduli obtained at each temperature are shifted to form a master curve by multiplying frequency by a factor *a_T_* (horizontal shifting) and allowing for a modest vertical shift with a factor *b_T_*. As an example, the full time‐temperature superposition process for a dissociative hydrogel (R = −0.5) is described in Figure S5 together with the curves obtained when *a_t_* is systematically maximized or minimized. We use this range in Figure 4B to quantify the errors on *a_T_*. Note that we systematically present temperature‐dependent properties obtained upon cooling rather than heating, in order to avoid history‐dependent effects. Indeed, although the values of a_t_ obtained upon heating and cooling are relatively close (Figure S5F), we always observe some small hysteresis, particularly near the transition temperature around 60°C.

## Funding

This project has received financial support from the Agence Nationale de la Recherche through the JCJC program Grant Number ANR‐20‐CE06‐0019 and from the CNRS through the MITI interdisciplinary programs Grant Number 249744.

## Conflicts of Interest

The authors declare no conflict of interest.

## Supporting information




**Supporting File**: adma72226‐sup‐0001‐SuppMat.docx.

## Data Availability

The data that support the findings of this study are available from the corresponding author upon reasonable request.
