# Supplementary material for: Decoupling Dynamics and Crosslink Stability in Supramolecular Hydrogels Using Associative Exchange
Source: Adv Mater. 2026 Jan 22;38(21):e16741. doi: 10.1002/adma.202516741 (PMC13073121; doi:10.1002/adma.202516741)
Supplement: Supplementary file 1 — Supporting File: adma72226‐sup‐0001‐SuppMat.docx. [file ADMA-38-e16741-s001.docx]

Supporting Information

Decoupling Dynamics and Crosslink Stability in Supramolecular Hydrogels Using Associative Exchange.

Pierre Le Bourdonnec, Charafeddine Ferkous, Léo Comunale, Luca Cipelletti, and Rémi Merindol*.


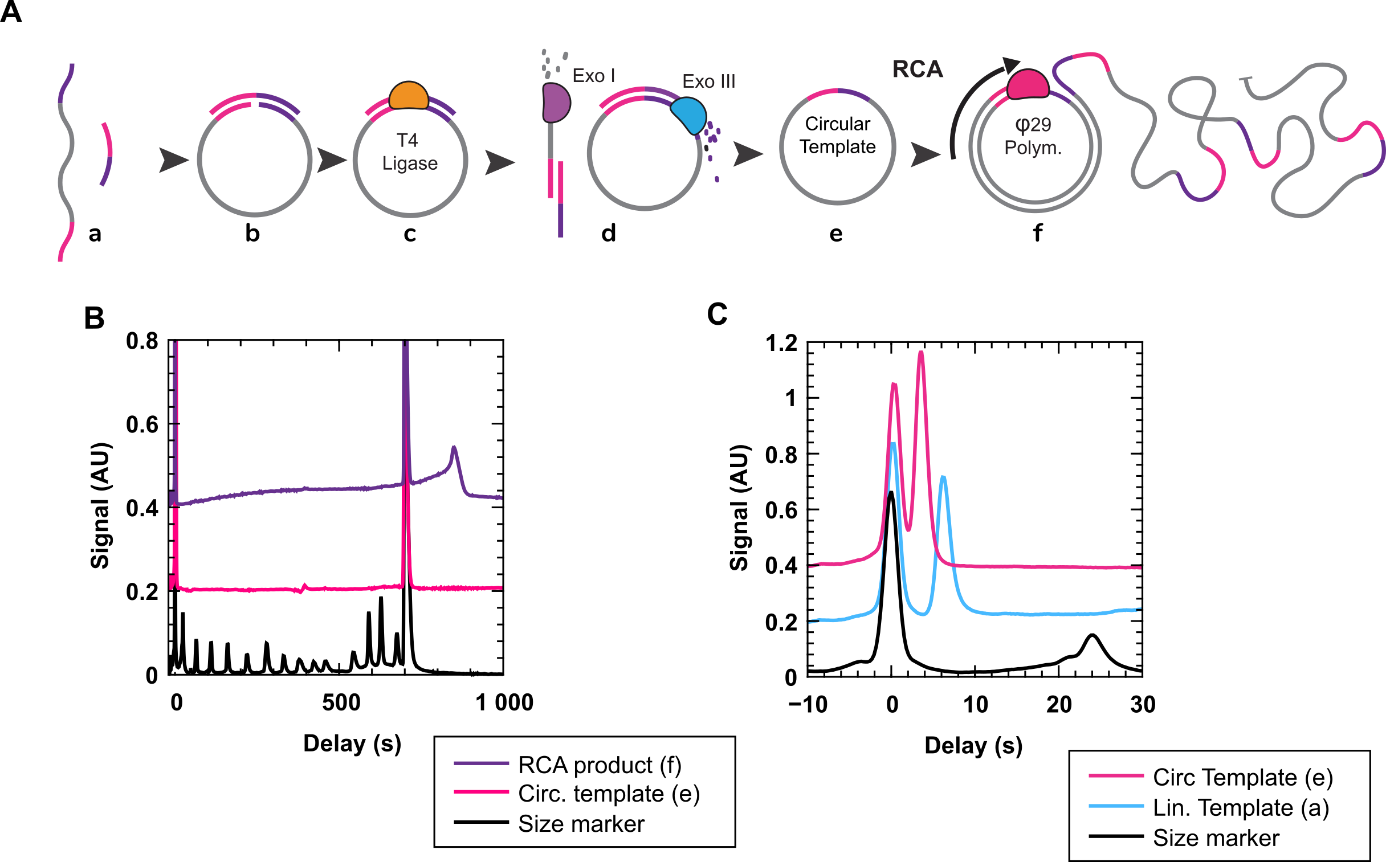


**Supporting Figure S1: DNA synthesis.** A) Schematic representation of the steps involved in the rolling circle amplification process. B) Representative electropherogram obtained via capillary gel electrophoresis, showing the circularized template (pink), the RCA product (purple), and the commercial DNA ladder (black) (From Bioptic Inc., Ref.109300, fragment sizes : 50, 100, 150, 200, 250, 300, 400, 430, 450, 500, 750, 1100, 1800 , and 3000 base pairs). The two peaks at 0 and 700s correspond to the alignment markers at 20 and 5000 base pairs, respectively. Data have been vertically offset for clarity. Due to the large time scale, the peak corresponding to the circular template is too close to the alignment marker to be distinguished. The RCA product, in contrast, displays a broad peak centered around 800s, beyond the 5000 base pair alignment marker. C) Zoomed view of the first 30s of the capillary electrophoresis signal, highlighting the peak of the circularized template (pink, at 4s) which appears slightly before the linear template peak (blue, at 8s).


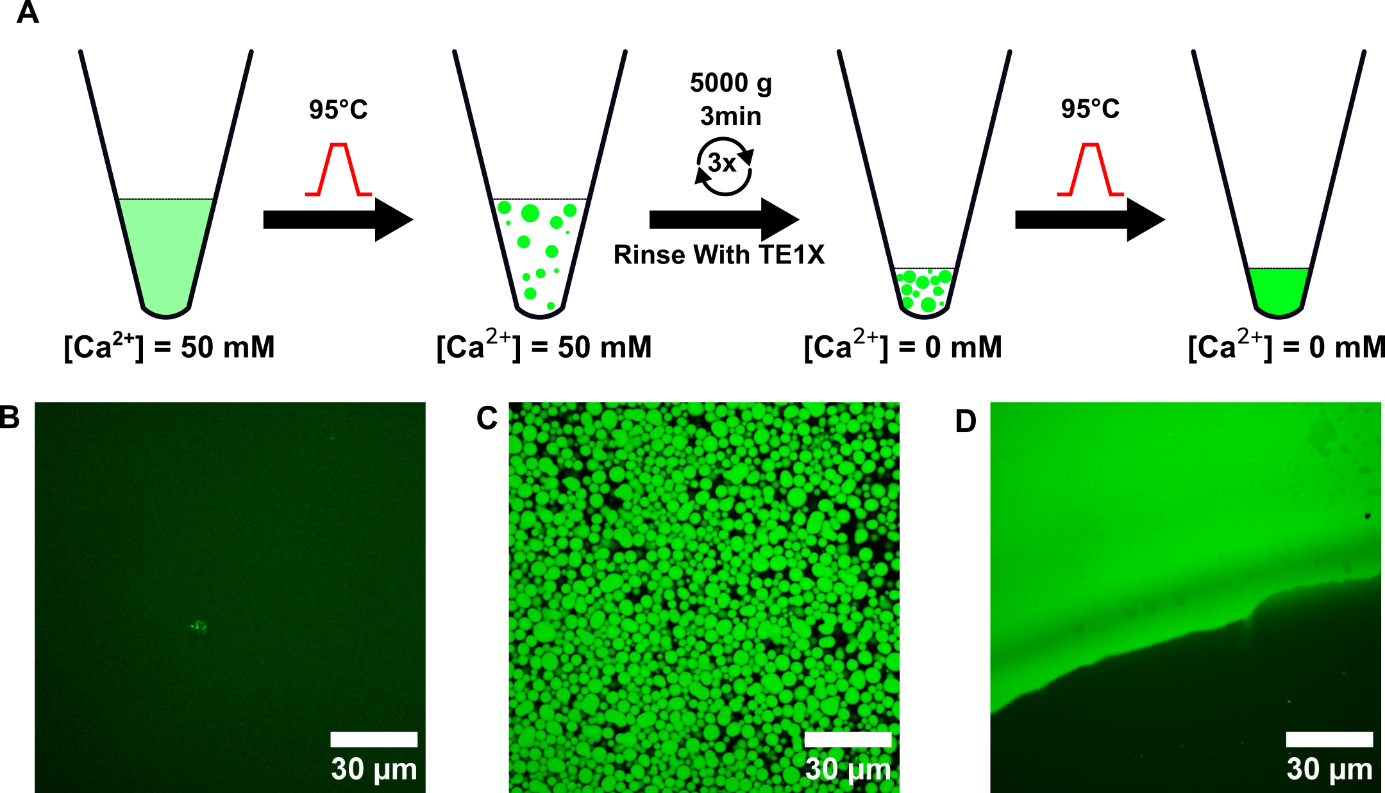


**Supporting Figure S2: Hydrogel assembly. A) Schematic representation of the steps involved in the hydrogel assembly process. B–D) Confocal microscopy images of an Atto_488_-labeled RCA product: as synthesized (B), after phase separation (C), and after hydrogel formation (D).**

**Supporting Note S1: Preparation of fluorescent DNA hydrogels.** The synthesis of fluorescent RCA products follows the protocol described in the main text, with the addition of 2 μL of fluorescent dNTP (10 mM; Aminoallyl-dUTP-XX-ATTO-488 or Aminoallyl-dUTP-XX-ATTO-594) to the dNTP mix. The hydrogel assembly protocol remains unchanged, except that 30% of one RCA product (typically sequence A) is replaced by a fluorescently labeled counterpart of the same sequence prior to microgel formation. For hydrogel melding experiments, spherical hydrogels are formed by introducing 3 μL of DNA microgel suspension (20 g·L⁻¹) into mineral oil preheated at 95 °C, incubating for 3 minutes, and then cooling to room temperature. After cooling, the spherical microgels can be manipulated using tweezers.

The microgels are manually inserted into a 96-well glass-bottom plate filled with fresh mineral oil, and pairs of microgels are gently brought into contact using tweezers. The plate is stored in a thermostated oven at 30 °C for 3 days. Imaging is performed at regularly spaced times and at room temperature, using a Leica DM8 wide-field fluorescence microscope with GFP and RFP filter sets.

**Supporting Note S2: Length of the RCA Products**

We reported in a previous article that DNA degrades at high temperature.^[1]^ Here, we take advantage of this process to control the size of the RCA products. Reducing the size of the RCA products is necessary to facilitate handling by lowering the viscosity of the strand suspensions, although it also impacts the resulting physical properties. Using capillary gel electrophoresis, we confirm that heating the RCA product in TE buffer at 95 °C decreases its molecular weight (Figure S3A). Initially, the product is so viscous that it does not migrate in the gel and shows no significant signal. After 5 minutes at 95 °C, a broad peak appears and progressively shifts to shorter migration times as the heating duration increases, indicating the formation of shorter DNA strands. This behavior clearly signals the thermal shortening of the RCA product. After 30 minutes at 95 °C, oscillations emerge in the electropherogram, corresponding to the repeat units of the RCA product. These oscillations suggest that cleavage preferentially occurs at specific sites within the sequence.

As we aim to form hydrogels from long DNA strands rather than short oligonucleotides, we focus on short heating times between 0 and 30 minutes at 95 °C. The loss and storage moduli of these hydrogels, measured as a function of heating time, are displayed in Figure S3B. The elastic modulus systematically decreases with increasing heating time, while the loss modulus remains nearly constant and only begins to decrease after 20 minutes of heating. This trend is expected, since shorter DNA strands form fewer entanglements and possess more free dangling ends that do not contribute to the elasticity of the network. Given the high polydispersity of the RCA product under all conditions, we do not explore this effect further. In all experiments, the RCA product undergoes heating for approximately 20 minutes at 95 °C, which ensures both ease of handling and reproducible mechanical behavior.


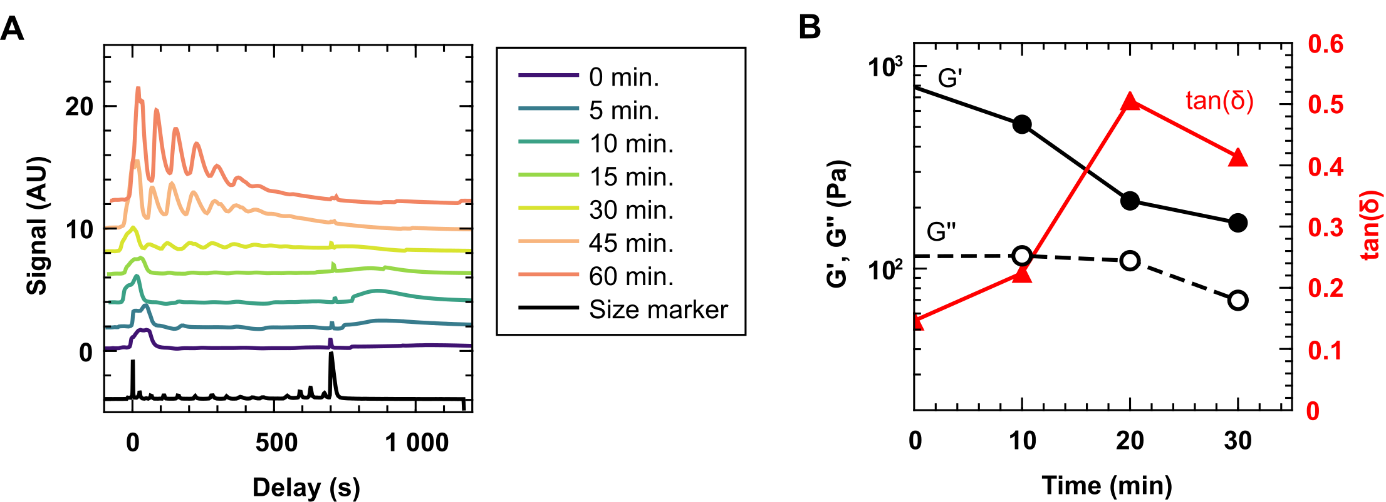


**Supporting Figure S3:** A) Electropherogram of RCA product A after 0 to 60 minutes in TE buffer at 95 °C. The size of the RCA product progressively decreases with heating time. The alignment markers are 20 and 5000 base pairs, and the size marker (Bioptic Inc., Ref. 109300) includes fragments of 50, 100, 150, 200, 250, 300, 400, 430, 450, 500, 750, 1100, 1800, and 3000 base pairs. B) Effect of heating time on the linear mechanical behavior of an R = -1 hydrogel at 1.5 wt%.

****Supporting Note S4:**** Fitting stress relaxation

Stress relaxation experiments are fitted with a stretched exponential decay, Equation 2 of the main text that we recall here for simplicity:

$\sigma\left( t \right)=\gamma G\left( t \right)+s= {\gamma G}_{0}\exp\left[ -\left( \frac{t}{\tau_{r}} \right)^{\beta} \right]+s$, Supporting Equation S1

where the fitting parameters are *G*_0_, *τ_r_*, *β*, and *s*. While in principle *s* = 0, in some cases we observed a small and constant remaining stress (<2 Pa) at long timescale. Its effect is negligible on static hydrogels (R≤0) as we do not observe the full relaxation. However, for dynamic hydrogels, this residual stress systematically leads to an underestimation of the stretching exponent β. We assumed this remaining stress to be an artifact and allowed for a small baseline correction (*s* <2 Pa) in the fits. Note that in Figure 3C of the main text we show the raw *G*(*t*)/*G*_0_ data without baseline correction, from which one can appreciate that such correction is indeed very small.

From the fitted *G*(*t*), the integral relaxation time is calculated as

$$\tau_{i}\left( G_{0},\tau_{r},\beta\right)=\frac{\int_{0}^{\infty} G\left( t \right)dt}{G_{0}}=A\int_{0}^{\infty} \exp\left[ -\left( \frac{t}{\tau_{r}} \right)^{\beta} \right]dt= A\tau_{r}\frac{\Gamma\left( 1/\beta\right)}{\beta}$$

Supporting Equation. S2

where *Γ*(*x*) is the Gamma function that generalizes the factorial function to non-integer arguments. Note that in writing Equation S2 we have explicitly included the dependence on *G*_0_: while *A* = 1 by definition (compare Equation S2 to Equation S1), this prefactor is affected by an uncertainty *δA* = *δG*_0_/*G*_0_, due to the error *δG*_0_ on the fitting parameter *G*_0_. The error bars on *τ*_i_ shown in the main text correspond to the propagation of the errors on *G*_0_, *τ_r_ , β*, obtained from the fits. For a set of fitting parameters $\bar{G}_{0},\bar{\tau}_{r}, \bar{\beta}$, one has (Equation S3) :

$$\delta\tau_{i}=\sqrt{\left[ \frac{\partial\tau_{i}}{\partial G_{0}} \right]_{\bar{G}_{0},\bar{\tau}_{r}, \bar{\beta}}^{2}\delta^{2}G_{0}+\left[ \frac{\partial\tau_{i}}{\partial\tau_{r}} \right]_{\bar{G}_{0},\bar{\tau}_{r}, \bar{\beta}}^{2}\delta^{2}\tau_{r}+ \left[ \frac{\partial\tau_{i}}{\partial\beta} \right]_{\bar{G}_{0},\bar{\tau}_{r}, \bar{\beta}}^{2}\delta^{2}\beta}$$

$= \sqrt{\frac{\delta^{2}G_{0}}{G_{0}^{2}}+\left[ \frac{\Gamma\left( 1/\beta\right)}{\beta} \right]_{\bar{\beta}}^{2}\delta^{2}\tau_{r}+ \left[ \frac{\partial\tau_{i}}{\partial\beta} \right]_{\bar{G}_{0},\bar{\tau}_{r}, \bar{\beta}}^{2}\delta^{2}\beta}$

Supporting Equation. S3

where the derivative of *τ_i_* with respect to *β* is calculated numerically.


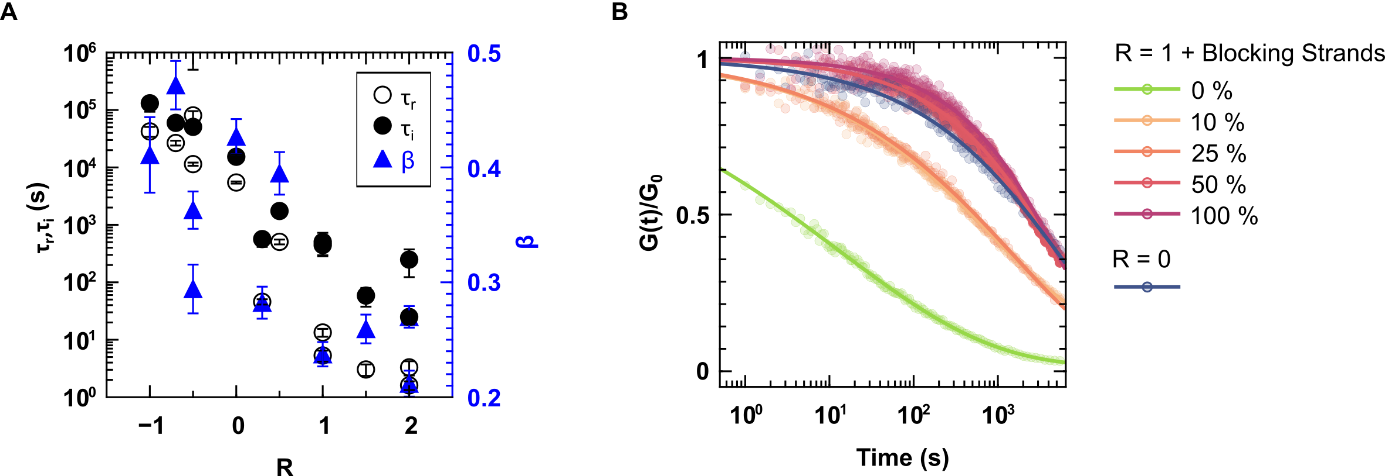


**Supporting Figure S4:** A) Values of the *1/e* relaxation time (*τ_r_*), integral relaxation time (*τᵢ*) and stretching exponent (*β*) obtained from fitting the stress relaxation data using a stretched exponential decay. B) Stress relaxation experiments for associative hydrogels (*R*=1) with increasing proportion of blocking strands (5% strain, 37 °C) and for dissociative hydrogels (*R*=0). Symbols represent experimental stress measurements; solid lines correspond to stretched exponential fits.


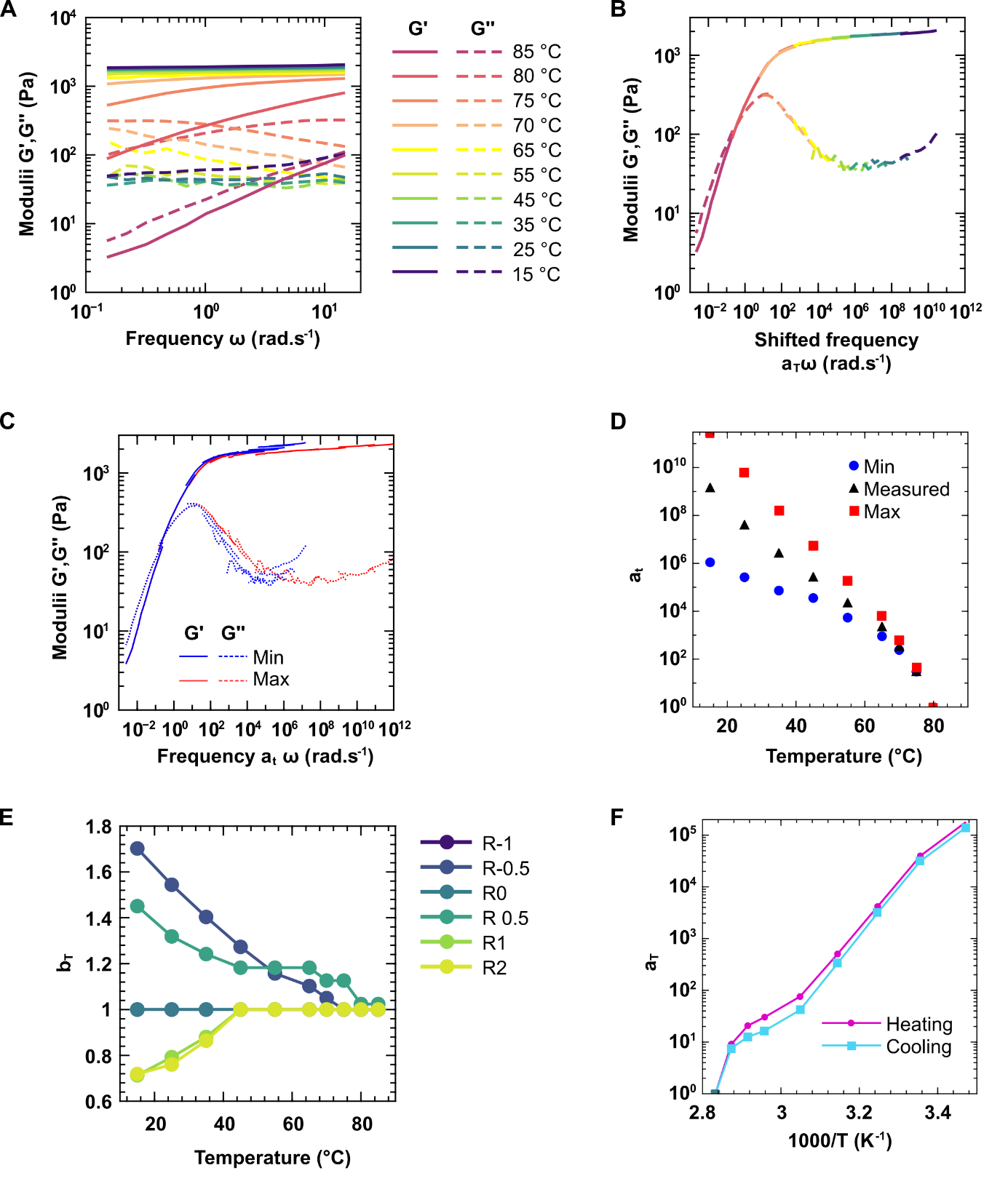


**Supporting Figure S5: Time Temperature Superposition** A) Evolution of the storage modulus (*G′*) and loss modulus (*G″*) obtained from frequency sweeps between 0.1 and 10 rad·s⁻¹ for a static hydrogel (*R* = -0.5) at temperatures ranging from 85 °C to 15 °C. B) Corresponding master curve obtained after manual alignment of all frequency sweeps. C) Examples of master curves that are overshifted (red) or undershifted (blue). Note that at low temperatures, the moduli are relatively insensitive to changes in both frequency and temperature, which complicates accurate superposition. D) Corresponding shift factors *aₜ* measured for the actual shift (values reported in the main text), as well as for overshifted and undershifted curves. As expected, a broad range of *aₜ* can be used to superimpose the curves of such a static hydrogel at low temperature. E) Values of the vertical shift bₜ applied to obtain the master curves. F) Examples of shift factors obtained either by heating (pink line) or by cooling (blue line) the sample.

****Supporting Note S5:**** Molecular Kinetics by Fluorometry

**Experimental design:** We aim to model the associative crosslink exchange and monitor the strand displacement reaction at the molecular scale. To achieve this, we construct a model strand displacement reaction using commercially synthesized DNA oligomers, tracked quantitatively via fluorescence labeling. The model reaction, shown in Figure S6A, consists of two parts: a background strand displacement reaction, which mimics the associative crosslink exchange, and a reporter reaction, which enables fluorescence-based monitoring of this exchange. To avoid confusion with the RCA products used in the hydrogel assembly, all short commercial oligomers used in this experiment are labeled with the prefix "x". In the background reaction, the oligomers xA, xB_1_, and xB_2_ share the same sequences as the RCA products A, B_1_, and B_2_, respectively, with the domains α, β_1_, β_2_ on A; α*, β_1_*, δ_1_ on B_1_; and α*, β_2_*, δ_2_ on B_2_. However, they lack the A_20_ spacer domain and consist of a single repeat unit. These sequences can be mixed at the same stoichiometric ratio *R* as in the RCA-based system, but they do not form a percolating 3D network. The reporter reaction involves two modified oligomers: xA_Q_, which has the same sequence as xA and is functionalized with a quencher (QXL_570_) at its 3′-end; and xB_1F_, which has the same sequence as xB_1_ and is labeled with a fluorophore (Atto_565_) at its 5′-end. When xA_Q_ and xB_1F_ hybridize, the fluorophore and quencher are brought into close proximity. In this configuration, the complex is non-fluorescent due to Förster Resonance Energy Transfer (FRET), whereby the fluorophore de-excites via energy transfer rather than photon emission. If xB_1F_ is displaced by xB_2_, the fluorophore becomes spatially separated from the quencher, FRET efficiency drops, and fluorescence increases. Because of the high efficiency of FRET, this system functions as a binary ON/OFF fluorescence sensors for the xA_Q_/xB_1F_ duplex. The fluorescence increase is therefore proportional to the loss of the xA_Q_/xB_1F_ complex, and to the formation of free xB_1_ and new duplexes xA/xB_1F_, both of which are fluorescent. Assuming that the duplexes xA_Q_/xB_1F_, xA/xB_1_, and xA/xB_2_ share similar reaction kinetics, and that the addition of a small amount of reporter does not perturb the global equilibrium, we can quantify the exchange kinetics of the background reaction by monitoring the fluorescence increase immediately after adding a small amount of reporter complex.

**Protocol.** The background reaction is prepared by mixing oligomers xA, xB_1_, and xB_2_ at the target stoichiometric ratio *R*, maintaining a constant total concentration of duplexes (xA/xB) at 2 µM in 400 µL of TENaMg buffer (10 mM Tris, 1 mM EDTA, 100 mM NaCl, 10 mM MgAc₂).

Separately, we prepare the fluorescent reporter by mixing xA_Q_ and xB_1F_ in a 1:1 molar ratio to form a duplex at 0.5 µM final concentration. Both the background and reporter mixtures are heated at 85 °C for 5 minutes, then cooled and equilibrated at the target temperature (between 15 °C and 55 °C) before starting the reaction. To initiate the fluorescence assay, we first add 8 µL of the reporter duplex to a 30 µL fluorescence cuvette (Hellma Analytics), which is then placed in a temperature-controlled fluorimeter (Cary Eclipse, Agilent, equipped with a Peltier temperature controller). We begin fluorescence acquisition with excitation at 565 nm, emission at 590 nm, and a recording interval of 0.1 s. After a few seconds, we add 200 µL of the thermalized background mixture into the cuvette and mix rapidly by pipetting the solution up and down five times. Fluorescence is then recorded for 3000 s at constant temperature. We determine the maximum theoretical fluorescence (*F_max_*) independently by measuring the fluorescence of a similar mixture (reporter + background) after incubation at 85 °C for 5 minutes, ensuring full equilibration. This measurement is performed at 25 °C.

For experiments involving long RCA products (reported in Figure 4C of the main text), we replace the background oligomers (xA, xB_1_, xB_2_) with the corresponding RCA products (A, B_1_, B_2_) at the same molar concentration of repeat sequence.

**Data treatment.** Typical fluorescence time traces (normalized by *F_max_*) are shown in Figure S6B as a function of the stoichiometric ratio *R*, and in Figure S6C as a function of temperature. The reaction is designed to follow first-order kinetics, since the concentration of displacing strand xB_2_ is much higher than that of the reporter complex xA_Q_/xB_1F_. Therefore, we expect the fluorescence *F(t)* to follow:

$F(t)=F_{max}*(1-e^{- \frac{t}{k_{app}}})$ Supporting Equation S4

where *t* is time, and *k_app_* is the apparent exchange rate, which depends on the concentration of available xB_2_ (thus on the stoichiometric ratio *R* of the background reaction). We extract k_app_ by plotting *ln(F_max_−F(t))* and fitting the initial slope of the curve (Figure S6D). Deviations from linearity at longer times are expected, as *F_max_* is determined independently and may vary due to sequence impurities or pipetting errors. This first-order kinetic assumption holds only if strand displacement is the sole mechanism of xA_Q_/xB_1F_ dissociation. At high temperatures, where DNA duplexes can thermally melt, this assumption breaks down. To validate the temperature range, we use an *R* = 0 background reaction and monitor fluorescence as a function of temperature. As shown in Figure S6E, the normalized fluorescence increase remains below 10% up to 55 °C. We therefore set 55 °C as the upper limit for our fluorescence experiments.

**Blocking strands.** We confirm in Figure S6F that blocking strands can inhibit the associative strand exchange. In this control, we compare an *R* = 1 background reaction with and without blocking strands. For the blocking condition, we add 22 µL of a mixture containing 20 µM each of Block-B_1_ and Block-B_2_ (in TENaMg buffer) 100 seconds after the start of the experiment. This results in a final blocking strand concentration of approximately 4 µM, corresponding to 100% saturation of available binding sites. Upon addition, the fluorescence increase slows down dramatically, indicating that the blocking strands efficiently inhibit strand exchange. In contrast, in the control reaction without blockers, fluorescence continues to increase steadily.


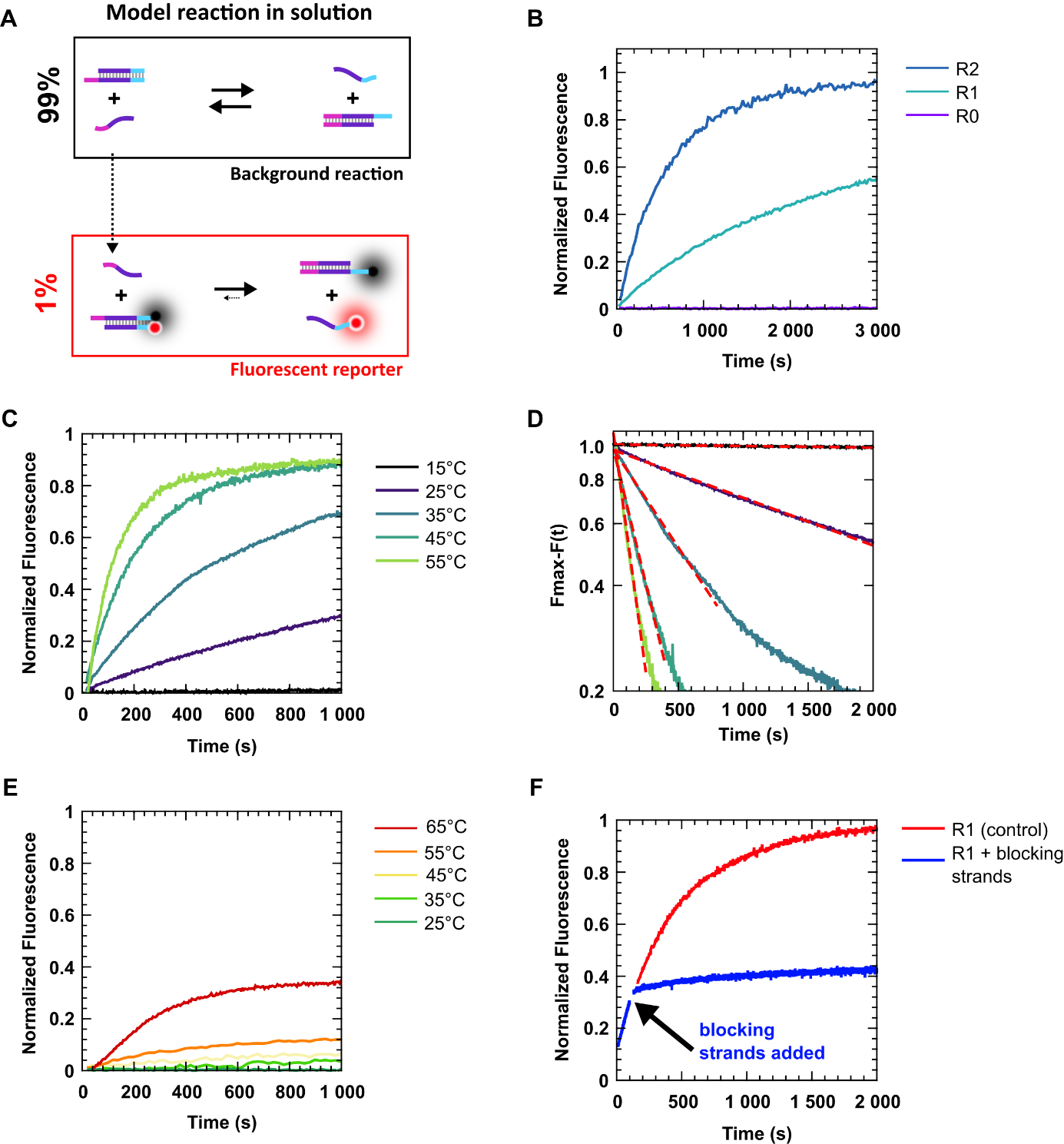


**Supporting Figure S6:** (A) (same as Fig. 4D of the main text) Schematic representation of the model strand displacement reaction used to monitor the kinetics of exchange in solution. The black box shows the background reaction, which consists of a mixture of unlabeled DNA oligomers (xA, xB_1_, xB_2_) mimicking the crosslinking domains of RCA products A, B_1_, and B_2_ (same sequences but without the A_30_ spacer). The red box shows the fluorescent reporter, a duplex formed by xA_Q_ (quencher-labeled) and xB_1F_ (fluorophore-labeled). Upon the displacement of xB_1F_ by xB_2_, the fluorophore separates from the quencher, stopping FRET and resulting in an increase in fluorescence. B) Evolution of fluorescence with time after adding the fluorescent reporter at *t* = 0, measured at 25 °C, for different stoichiometric ratios *R* of the background reaction. C) Evolution of fluorescence with time after adding the fluorescent reporter at *t* = 0, measured at different of temperatures for a constant stoichiometric ratio *R* = 1 for the background reaction. D) Examples of the linear fits (dashed red lines) of the plots *ln(F_max_−F(t))* versus *t*, used to extract the apparent exchange rate *k_app_*, assuming first-order kinetics. The data correspond to the same experiments and color code as in (B). E) Control experiments showing a negligible fluorescence increase below 55 °C for a background reaction stoichiometric ratio *R* = 0, confirming that the strand displacement reaction is negligible under these conditions. F) Validation of strand exchange inhibition by blocking strands. Upon addition of 100% blocking strands at t = 100 s in a reaction with *R* = 1, the fluorescence increase slows dramatically, confirming efficient suppression of the exchange. In contrast, the control without blockers continues to exhibit fluorescence growth.

**Supporting Note S6: Linking Activation Energy to Thermodynamic Parameters**

Discussing activation energies from a thermodynamic perspective requires some care. For a single step reaction, the activation energy (*Eₐ*) is determined by the free energy barrier of the transition step (i.e., *Eₐ = ΔG^‡^*). The lower the *ΔG^‡^*, the faster the reaction at a given temperature. However, since *ΔG^‡^* itself varies with temperature, the slope of the curves in an Arrhenius plot is not directly proportional to *ΔG^‡^*. By decomposing the free energy variation *ΔG^‡^* into its temperature-independent thermodynamic components, enthalpy (*ΔH^‡^*) and entropy (*ΔS^‡^*), we obtain the Eyring–Polanyi equation (**Supporting Equation S5**).

$a_{T}\propto A\cdot e^{\left( -\frac{{\Delta G}^{\ddagger}}{R_{g}\cdot T} \right)}=A\cdot e^{\left( -\frac{{\Delta H}^{\ddagger}-{T\Delta S}^{\ddagger}}{R_{g}\cdot T} \right)}=A{\cdot e^{\left( -\frac{{\Delta S}^{\ddagger}}{R_{g}} \right)}\cdot e}^{\left( -\frac{{\Delta H}^{\ddagger}}{R_{g}\cdot T} \right)}$ **Supporting** Equation S5

From Supporting Equation S5, we see that the slope of the Arrhenius plot (i.e. the activation energy), corresponds to the enthalpy change *ΔH^‡^* of the transition step. Nevertheless, it is the free energy barrier that ultimately determines the absolute rate of a reorganization mechanism.

This analysis is not strictly applicable to multistep processes, because the existence of different steps can impact the concentration of reacting species and hence the rate of the limiting step. However, if one of the steps is significantly slower than the others, it will effectively act as a limiting step that determines the overall rate of the multistep reaction.^[2,3]^ We believe that such an approximation may apply to the case of dissociative and associative crosslink exchange. For dissociative exchange, we propose that the rate-limiting step is the duplex melting, while for the associative process it is the initiation of invasion. Bearing in mind these hypotheses, we compare quantitatively the enthalpy variation (*ΔH)* predicted for these rate-limiting steps (Table S1) with the slopes measured in the Arrhenius plot. On the one hand, the values predicted for the dissociative reaction pathway (*ΔH_S0→S1_*= 735 kJ/mol) match those found for dissociative hydrogels. On the other hand, for the associative exchange, the predicted activation energy, *ΔH_S2→S3_* = 88 kJ/mol, is lower than the value obtained from TTS, *Eₐ* = 165 ± 20 kJ/mol, but it does match the activation energy of 90 ± 15 kJ/mol measured using short oligomers in solution.

In the intermediate regime (observed in the TTS analysis between 55 °C and 75 °C), where the rate-limiting step becomes the direct invasion S_0_ → S_3_ of the duplex (Supporting Figure S7), we expect a negative enthalpy variation (ΔH_S0→S3_ = –88 kJ·mol⁻¹, see Supporting Table S1). Given that these enthalpy estimates are based on oligomers in solution and do not account for contributions from the network, this value appears reasonable and helps rationalize the plateau observed in the Arrhenius plot (Figure 4B). Overall, despite the fact that strand-displacement reactions are multistep processes, the activation energies measured experimentally seem to correlate with the calculated enthalpy variations of the corresponding rate-limiting steps.


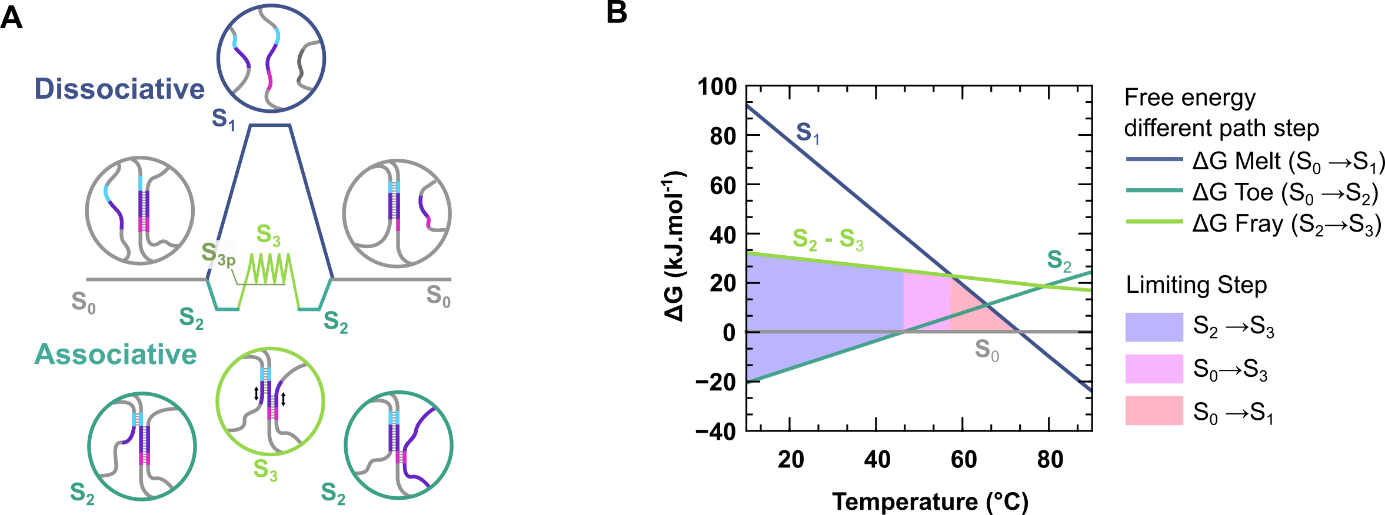
 **Supporting Figure S7: Thermodynamic profiles** A) Schematic thermodynamic profile of dissociative (blue) and associative (green) crosslink exchange pathways, including the names and molecular representations of the states. B) Temperature-dependent evolution of the free energy levels (*ΔG*) for the various assembly states. Values are calculated relative to S_0_ using theoretical thermodynamic parameters for oligomers in solution (see Table S1). The shaded regions indicate the rate-limiting steps for crosslink exchange in an associative hydrogel (*R* > 0). Three colors highlight distinct regimes: at low temperature (blue), the limiting step is the strand invasion following toehold hybridization (S_2_ → S_3_); at intermediate temperatures (pink), it is the direct invasion without toehold hybridization (S_0_ → S_3_); and at high temperature (red), it is the melting of the duplex during dissociative exchange (S_0_ → S_1_).

**Supporting Note S7: Theoretical thermodynamic profiles**.

The thermodynamic properties of DNA hybridization are well established.^[4]^ Here, we employed the UNAFold platform (https://www.unafold.org) to compute the theoretical thermodynamic parameters for hybridization of the various DNA domains.^[5,6]^ For these simulations, we set the NaCl concentration to 100 mM and the concentration of each DNA oligomer to 400 µM, corresponding to the concentration of repeat units in a 2 wt% hydrogels. Since UNAFold does not support strand displacement reactions, we incorporated additional thermodynamic parameters from literature. The free energy profile of the strand displacement has been extensively described by Winfree and coworkers in their study on strand displacement kinetics.^[7]^ They reported a total free energy change of 30 kJ·mol⁻¹, decomposed into two components: (1) an initiation energy barrier, associated with the formation of a three-stranded junction, corresponding to a plateau height of *ΔG_p_ =* 8 kJ·mol⁻¹, and (2) an energy cost for progressive base-pair unzipping during junction migration, corresponding to a sawtooth pattern of *ΔG_s_* = 22 kJ·mol⁻¹, as illustrated in Figure S7A (green line, states S3p and S3 respectively). To model the temperature dependence of the energy profile (see Figure 4E, in the main text), we further decomposed the free energy (*ΔG*) into enthalpic (*ΔH*) and entropic (*ΔS*) contributions. For the plateau, we used Winfree’s estimated values from their Supplementary Information (page 13): *ΔHₚ* = 54 kJ·mol⁻¹ and *ΔSₚ* = 154 J·mol⁻¹·K⁻¹.^[7]^ For the sawtooth component, no direct values for *ΔH* and *ΔS* are available in the literature. To estimate these parameters, we referred to the unified nearest-neighbor thermodynamic model by SantaLucia,^[4]^ choosing GA/CT base pairs as a representative case with intermediate hybridization strength. For these, the free energy of hybridization is *ΔG_GA/CT_* ≈ 6 kJ·mol⁻¹. Notably, this value is significantly lower than the 22 kJ·mol⁻¹ calculated by Winfree for strand displacement. We hypothesize that this discrepancy arises from a reduced entropic gain during strand migration compared to DNA melting. In DNA melting, base-pair disruption results in strand separation, increasing system entropy via a greater number of accessible microstates. In contrast, during branch migration, base unstacking occurs between already hybridized duplexes, contributing little to the entropy change. Accordingly, we propose the following decomposition for the sawtooth free energy: *ΔHₛ* = 34 kJ·mol⁻¹ = *ΔH_S3p🡪S3_*, taken directly from SantaLucia,^[4]^ and *ΔSₛ* = 39 J·mol⁻¹·K⁻¹= *ΔS_S3p🡪S3_*, adjusted to yield *ΔGₛ* = 22 kJ·mol⁻¹ at 25 °C, under the assumption that branch migration proceeds via single base-pair rupture. The resulting thermodynamic data, used to construct the free energy diagrams in Figure 4D and Figure S7, are summarized in Table S1. The good agreement between the theoretical enthalpy variation and the activation energy derived from fluorometric measurements (Figure 4C) further supports this hypothesis.

| **Supporting** Table S1 : Thermodynamic Data. | | | | |
| --- | --- | --- | --- | --- |
|  | ΔH  (kJ.mol^-1^) | ΔS  (J.mol^-1^.K^-1^) | ΔG at 25°C  (kJ.mol^-1^) | Reference |
| S_0_  (Reference) | 0 | 0 | 0 | UNAFold, [Oligo] = 400 µM , [Na^+^] = 100 mM. |
| S_0_ 🡪 S_1_ | +735 | +2068 | +119 | UNAFold, [Oligo] = 400 µM , [Na^+^] = 100 mM. |
| S_0_ 🡪S_2_ | -176 | -552 | -12 | UNAFold, [Oligo] = 400 µM , [Na^+^] = 100 mM. |
| S_2_ 🡪S_3p_  (plateau height) | +54 | +155 | +8 | ^[7]^ |
| S_3p_🡪S_3_  (sawtooth amplitude) | +34 | +40 | +22 | ^[4,7]^ |
| S_2_ 🡪S_3_ | +88 | +195 | +30 | S_2_ 🡪S_3p_ + S_3p_ 🡪S_3_ |
| S_0_ 🡪S_3_ | -88 | -357 | +18 | S_0_🡪S_2_ + S_2_🡪S_3_ |

****Supporting Note S8:**** Poly-Lysine treatment and startup shear experiments

The effect of poly-D-lysine treatment is most evident in start-up shear experiments (Figure S8). Without treatment, the hydrogel breaks below 5 kPa (Figure S8B), while an hydrogel from the same batch, measured after treating the rheometer tools with poly-D-lysine, withstands stresses approaching 40 kPa. In the linear regime (below 100% strain), both curves overlap, confirming that poly-D-lysine treatment does not affect the bulk mechanical properties of the hydrogels. We also use fluorescently labeled DNA hydrogels and a UV lamp (365 nm, 6 W) to visualize the sample after mechanical rupture. Without poly-D-lysine treatment, the sample remains in one piece on the bottom plate of the rheometer, indicating detachment from the upper tool. With poly-D-lysine treatment, the sample fractures in the bulk into multiple pieces, with portions adhering to both rheometer surfaces after failure (Figure S8A). This result confirms that strong adhesion to the rheometer tools is necessary to measure the true rupture strength of DNA hydrogels. Start-up shear experiments are performed at a high strain rate (1 s⁻¹), which minimizes the effect of network reorganization on rupture behavior. After each test, the hydrogel is heated at 85 °C for 5 minutes to reform the network. The first rupture typically results in a higher ultimate stress than subsequent ruptures (Figure S8C), which yield consistent values. Therefore, we only report start-up shear data obtained after the first rupture.


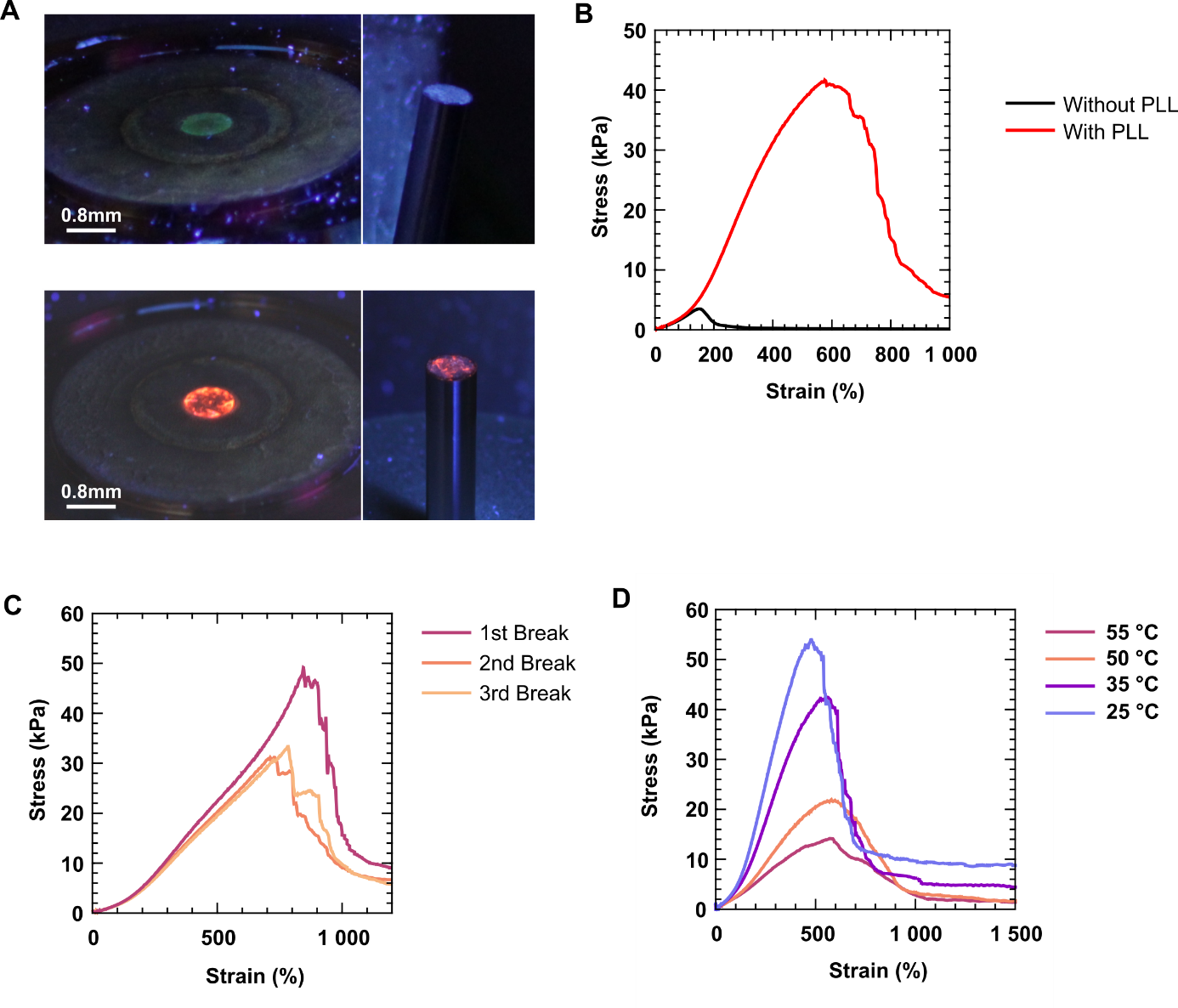


**Supporting Figure S8:** A) Photographs of fluorescent DNA hydrogels under UV illumination (365 nm, 6 W) after start-up shear experiments, shown without (top) and with (bottom) poly-D-lysine treatment. B) Effect of poly-D-lysine treatment on start-up shear experiments performed on an *R* = -1 hydrogel at a strain rate of 1 s⁻¹. C) Multiple start-up shear experiments performed on the same *R* = -1 hydrogel, with the network reformed at 85 °C between tests. D) Startup shear experiments on an associative hydrogel R=1 at different temperatures.

**References**

[1] R. Merindol, S. Loescher, A. Samanta, A. Walther, *Nat. Nanotechnol.* **2018**, *13*, 730.

[2] J. C. Polanyi, *Science* **1987**, *236*, 680.

[3] C. Stegelmann, A. Andreasen, C. T. Campbell, *J. Am. Chem. Soc.* **2009**, *131*, 8077.

[4] J. Santalucia Jr., *Proc. Natl. Acad. Sci. U.S.A.* **1998**, *95*, 1460.

[5] N. R. Markham, M. Zuker, *Methods Mol. Biol.* **2008**, *453*, 3.

[6] M. Zuker, *Nucleic Acids Res.* **2003**, *31*, 3406.

[7] N. Srinivas, T. E. Ouldridge, P. Sulc, J. M. Schaeffer, B. Yurke, A. A. Louis, J. P. K. Doye, Erik. Winfree, *Nucleic Acids Res.* **2013**, *41*, 10641.
